# Analysis of Clinical Efficacy of Traditional Chinese Medicine in Recovery Stage of Stroke: A Systematic Review and Meta-Analysis

**DOI:** 10.1155/2020/7172052

**Published:** 2020-09-19

**Authors:** Xue Zhang, Xiao-Fei Zhang, Lin Wang, Dong-Yan Guo, Jia-Min Zhang, Yong-Gang Chen, Zhi-Chao Wang, Li-Shan Pei, Jiang-Xue Chen, Ya-Jun Shi, Jun-Bo Zou

**Affiliations:** Shaanxi Province Key Laboratory of New Drugs and Chinese Medicine Foundation Research, Pharmacy College, Shaanxi University of Chinese Medicine, Xianyang, China 712046

## Abstract

**Background:**

We provide an updated meta-analysis with detailed information on a combination of TCM and routine treatment.

**Methods:**

Retrieve appropriate articles with no language restrictions on keywords until 8 July 2019 in an electronic database. All trajectories are screened according to certain criteria. The quality of certified research was also evaluated. We made a detailed record of the results of the measurement. Meta-analysis was carried out by using the Revman 5.3 software.

**Results:**

Sixty-seven RCTs were included, and 6594 subjects were analyzed. Compared with routine treatment, the total effective rate (TER) of TCM combined with routine treatment was improved, and the recovery of stroke was also significantly accelerated. Regulation of blood lipids by notably shrinking the contents of TC, TG, and LDL and enhancing the levels of HDL. The levels of serum hs-CRP, WHV, and WLV decreased significantly, indicating that the expression of thrombomodulin was decreased after the comprehensive treatment of traditional Chinese medicines (TCMs). The combination of TCM treatment could enhance the protection of neural function by decreasing the NIHSS scoring while increasing the BI scoring. Paeoniae Radix Rubra, Angeticae Sinensis Radix, etc., can effectively improve the clinical symptoms of stroke convalescent patients and promote the recovery of neurological function. ACU of Baihui, Renzhong, etc., can improve the clinical rehabilitation effect of patients. However, our findings must be handled with care because of the small sample size and low quality of clinic trials cited. Other rigorous and large-scale RCTs are in need to confirm these results.

**Conclusion:**

A combination of TCM and routine treatment in the treatment of stroke could improve TER, and it is beneficial to the rehabilitation of patients in the recovery period of apoplexy. These effects can be mediated by a combination of several mechanisms. Nevertheless, due to the limitations of this study, these results should be handled with caution.

## 1. Introduction

A stroke is an injury to a part of the brain that results in the death of brain cells which can be caused by a blockage of blood flow to a part of the brain (ischemic stroke) or by a tear of a blood vessel causing bleeding into the brain (hemorrhagic stroke) [1]. Stroke is highly prevalent and is one of the major contributors to morbidity and mortality worldwide [2]. The world is facing an epidemic of stroke [3]. Each year, stroke affects around 9 million people worldwide for the first time and results in long-term disability for around 6.5 million people [4]. Stroke is the leading cause of death in China and the second leading cause of death in the world [5]. In China, about 2.5 million people suffer from strokes each year, and 70% to 80% of patients lose the ability to carry out daily activities and routine care, resulting in a financial burden on the state and families [6]. Stroke is also the second leading cause of disability-adjusted life years globally [7].

Stroke not only impairs neurological function but also leads to severe medical complications [8]. Common deficits after stroke include weakness, numbness, vision problems, slurred speech and swallowing problems, difficulties with language, equilibrium and coordination problems, and problems with thinking [1]. They are terrifying ordeals that usually occur without warning—even though the causes are known—and rob people of their independence through impaired speech and movement [9]. The damage caused by a devastating stroke to individual patients and families is incalculable; most elderly patients fear a disabling stroke more than they fear death [10]. Therefore, strengthening the treatment of the stroke recovery period is the key to reduce the mortality and disability rate. However, modern medicine lacks effective treatment for its recovery period, while traditional Chinese medicine (TCM) has great superiority [11]. TCM is frequently used throughout the world for stroke patients [12]. The purpose of TCM in the treatment of stroke is to reduce the symptoms of patients and eliminate the underlying causes. With a long history of thousands of years, TCM plays an important role in the treatment of complex diseases worldwide [13]. Besides China, TCM is popular not only in other parts of Asia but also in some western countries including in the USA and Australia [14]. TCM has attracted much attention because of its unique theoretical bases, which is quite different from that of western medicine. TCM emphasizes the importance of using prescriptions, natural products, ACU, and physical exercise to improve the ability of individual endogenous healing through preventive, holistic, and healthy methods [15]. And the WHO has been avidly supporting traditional medicines, especially TCM, as a step towards its long-term goal of universal health care. According to the agency, in some countries, traditional treatments have the advantages of being cheaper and more accessible than western medicine [16]. TCM includes a wide range of practices, like herbal medicine and ACU, as well as other practices peculiar to most Westerners, such as cupping (heated cup therapy), tuina (massage), and moxibustion (burnt mugwort therapy) [17]. China's considerable experience in the use of TCMs (traditional Chinese medicines) in stroke treatment shows that TCM preparations are effective, with few or no side effects. Other studies have pointed out that TCMs have many targets and a wide range of ways of action, which is in accordance with the pathophysiological process of stroke. In TCM, more than 100 kinds of TCMs have been used to prevent and treat stroke [18]. ACU has been used as a medical modality for over 3000 years in China. ACU is often used as an aid to mainstream rehabilitation after stroke, including the insertion of ACU needles into the skin of certain parts of the body [19]. The basic principle of ACU treatment is that intervention at specific acupoints on meridians and collaterals related to a specific organ system can restore the proper energy balance in the body, thus restoring the patient to health [20]. Other treatment of traditional Chinese medicine (OTTCM) includes moxibustion, needle knife, acupoint catgut embedding, cupping, and scraping. To sum up, TCM has the merits of diminishing disability rate, boosting quality of life, low toxicity and side effects, and low therapy cost for patients in poststroke recovery.

**Figure 1:**
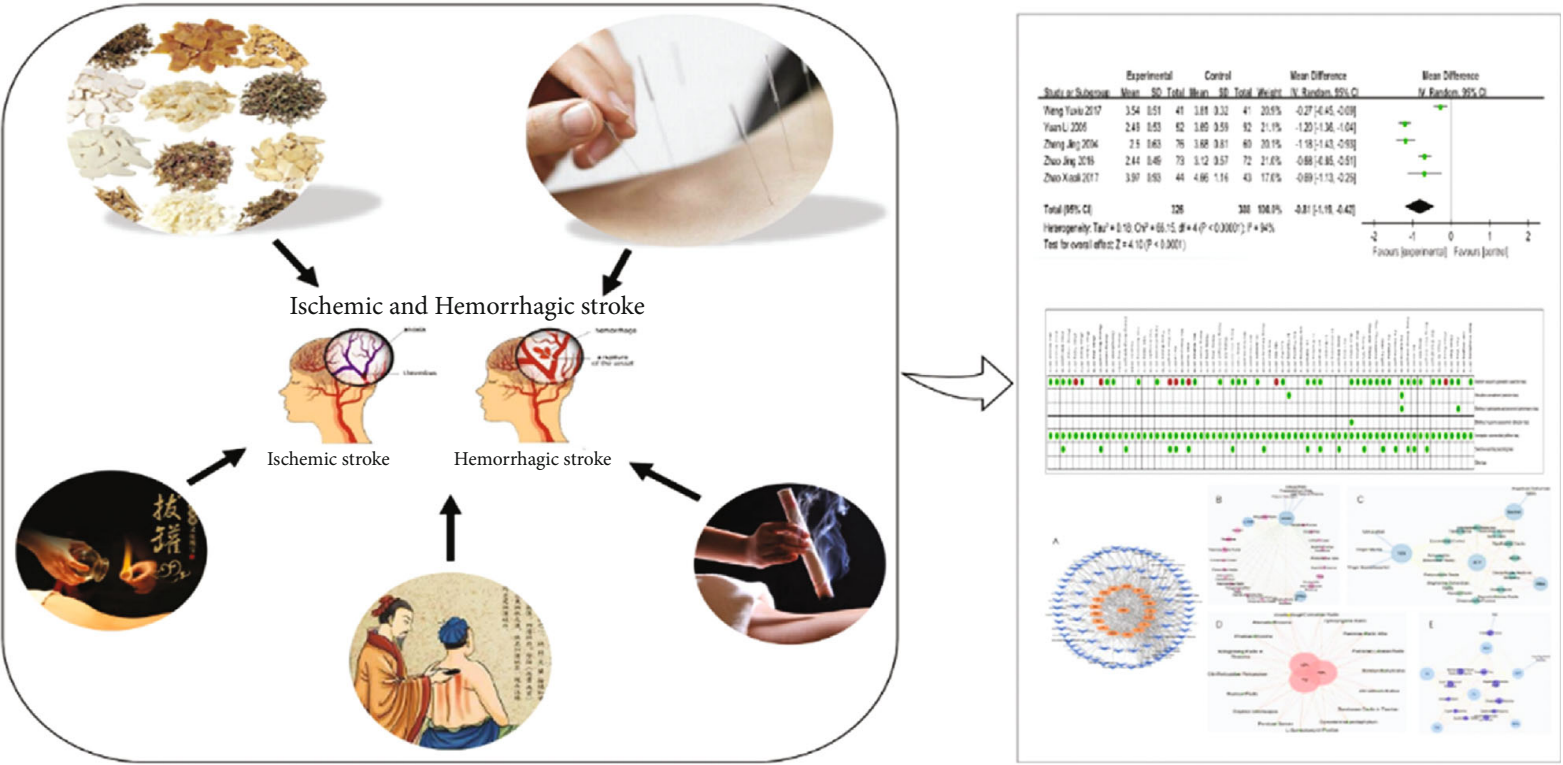
Workflow of the present study.

Despite numerous TCM interventions evaluated in previous randomized controlled trials (RCTs) to treat stroke, it is not comprehensive enough. Therefore, we have provided an updated and expanded meta-analysis, which provides detailed information for the combination of TCM and conventional treatment for stroke patients (Figure 1).

## 2. Methods and Program

### 2.1. Literature Retrieval Strategy

Keywords “stroke” or “Cerebral apoplexy” [Title/Abstract] AND “Clinical” [Title/Abstract] AND at least one of the following items including “Acupuncture” [Title/Abstract],“Traditional Chinese medicine” [Title/Abstract], “Moxibustion” [Title/Abstract], “Needleknife” [Title/Abstract], “Cupping” [Title/Abstract], “Scraping” [Title/Abstract], and “Traditional Chinese medicines” [Title/Abstract] were used as search items in electronic databases including PubMed, Wanfang, the China National Knowledge Infrastructure (CNKI), the VIP medicine information system (VMIS), Embase, the Cochrane Library, and the Chinese Biomedical Database (CBM), separately. All of the searches were performed from inception to July 2019. All relevant articles were downloaded into the EndNote software (version X9, Thomson Reuters, Inc., New York, NY, United States) for further exploration. A duplicate record was deleted. A full-text review was performed while the title/abstract was thought to be thematic. Three researchers independently assessed literature eligibility. Any disagreement was resolved by a consultation with a group discussion.

### 2.2. Inclusion and Exclusion Criteria

Based on the recommendations of the experts, we have designed the following inclusion criteria: (1) Patients in RCTs were diagnosed with stroke by the fourth National Conference on Cerebrovascular Diseases or criteria for diagnosis and evaluation of curative effect of apoplexy (CEECEA), or Guidelines for the Diagnosis of Acute Ischemic Stroke in China (GDAISC), or diagnostic criteria for midbrain infarction in neurology (DCMIN) version 7, or Diagnostic Essentials of all kinds of Cerebrovascular Diseases (DECD) version 1995, or criteria for diagnosis and evaluation of therapeutic effect of apoplexy in traditional Chinese medicine (CDETEA), or Guidelines for the Prevention and Treatment of Cerebrovascular Diseases of the Chinese Society of traditional Chinese medicine (GPTCDCS) version 2010. (2) All trials mentioned were described as RCTs. (3) The experimental group treated with TCM treatment is based on the control group, while the control group was only given routine treatment. (4) The measurement of the results of each study must include at least one of the following indicators: high-sensitivity C-reactive protein (hs-CRP), total cholesterol (TC), triglyceride (TG), low-density lipoprotein (LDL), high-density lipoprotein (HDL), plasma viscosity (PV), whole low viscosity (WLV), hematocrit (HCT), whole high viscosity (WHV), homocysteine (HCY), fibrinogen (FIB), National Institutes of Health Stroke Scale (NIHSS), Fugl-Meyer Assessment (FMA), Barthel Index (BI), clinical spasticity index (CSI), modified Rankin scale (MRS), Syndrome Integral of Traditional Chinese Medicine (SITCM), standardized swallowing assessment (SSA), videofluoroscopy swallowing study (VFSS), vascular endothelial growth factor (VEGF), evaluation result of activities of daily living (ADL), immunoglobulin A (IgA), immunoglobulin G (IgG), and immunoglobulin M (IgM).

If the study has one of the following items, it is not included: (1) Articles such as reviews, experiments, case reports, and missing data are considered to have nothing to do with the subject. (2) The trial is not an RCT, or the diagnostic criteria in the statement are not clear. (3) Intervention for stroke patients is not based on TCM treatment.

### 2.3. Data Extraction and Quality Assessment

Information about qualified studies including authors, sample size, year of publication, type of intervention, and outcome measures was extracted and arranged in the tables. The quality of inclusion in the study was independently assessed by three researchers based on the Cochrane Intervention System Review Manual. Disagreement was settled by the consensus. The quality assessment is as follows: random sequence generation (selection bias), allocation concealment (selection bias), blinding of participants and personnel (performance bias), blinding of outcome assessment (detection bias), incomplete outcome data (attrition bias), selective reporting (reporting bias), and other bias. Each semester is judged at three levels. The “low risk” of prejudice means that the description of the method or procedure is adequate. An inadequate or incorrect description of a method or procedure means “high risk,” while the absence of a description of a method or procedure means “unclear risk.” Two researchers used the GRADE system to grade the quality of evidence for all outcome indicators. Evaluation indicators include risk of bias, inconsistency, indirectness, imprecision, publication bias, large effect, plausible confounding, and dose-response gradient, a total of 8 factors, of which the first 5 are degrading factors and the latter 3 are escalating factors. The level of evidence is divided into four levels: high, moderate, low, and very low.

### 2.4. Data Analysis

Analyze the data by using Review Manager 5.3 (Cochrane Collaboration). Outcome measures such as TER were treated as dichotomous variables and emerged as the odds ratio (OR) with 95% confidence intervals (95% CI). Factors of blood lipid (TC, TG, LDL, and HDL), FMA scoring, NIHSS scoring, and so on were continuous variables that appeared the mean difference (MD) with 95% CI. We evaluated the heterogeneity between the studies by using *Q* statistics and *I*^2^ tests. The data with low heterogeneity (*P* ≥ 0.1% and *I*^2^ ≤ 50%) were analyzed by using a fixed-effects model, while the data with high heterogeneity (*P* < 0.1 or *I*^2^ > 50%) were estimated by using the random-effects model. Funnel plots reveal potential publication bias. Egger's test was further executed to examine the publication bias by meta for a package in R platform [21].

## 3. Results

**Figure 2:**
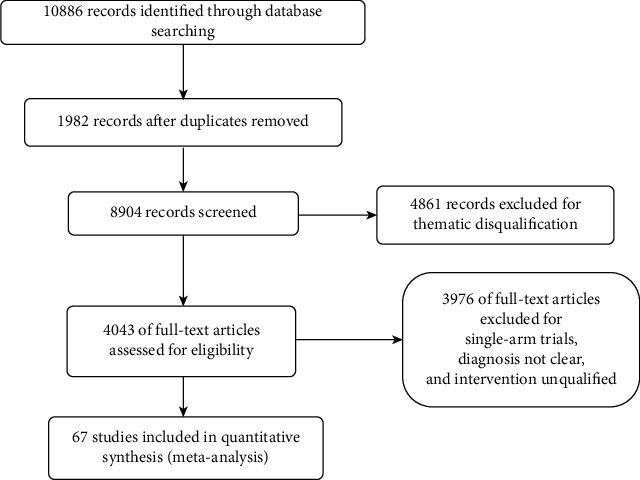
Process of study extracted for the meta-analysis.

After the database search, 10886 articles were identified, of which 1982 duplicate articles were deleted. Of the remaining 8904 articles, 4861 were excluded because of thematic disqualification. After the preliminary screening, there are still 4043 articles waiting for further full-text review. In the process, 3976 studies were excluded for the following reasons: (1) Diagnosis was vague. (2) There are mentioned unfit interventions. (3) There are single-arm designs. Finally, 67 studies [22–88] were included (Figure 2).

In this meta-analysis, 6544 patients with stroke were collected (3396 in the experimental group and 3198 in the control group). The patient's age ranged from 18 to 85 years, and there was no significant ff difference in age and gender between the two groups. The age of the patients was between 18 and 85 years old, but there was no substantial difference in age and sex between the two groups (Table 1). All trials were conducted between 2004 and 2019, all of which were RCT, and combined TCM with routine treatment and routine treatment. Routine treatment is slightly different in qualified trials, and the usual method is to give some conventional anticoagulant, anti-infection, control blood pressure, control water and electrolyte disorders, and other drugs. Sixty-seven studies reported the duration of treatment lasted for 12 months. Six trials reported a follow-up ranging from 1 to 12 months (Table 2). At the same time, we also mentioned the prescription of TCM and its origin (Table 3), the composition of acupoints (Table 4), and the treatment scheme of the ACU control group (Table 5). We also provide international codes for acupoints (see optional Supplementary Materials (available here). Using the GRADE system to grade the quality of evidence for all outcome indicators, the results of GRADEpro showed the quality of the evidences of 2 outcomes was high, 13 outcomes were moderate, 9 outcomes were low, and 2 outcomes were very low (see optional Supplementary Materials).

### 3.1. Quality of Included Trial Assessment

**Figure 3:**
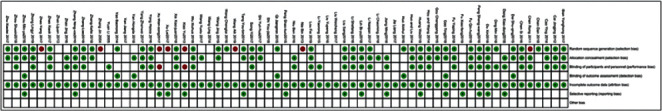
Risk of bias assessment in eligible studies. The quality assessment was conducted by Review Manager 5.3 according to the Cochrane Handbook for Systematic Reviews of Interventions Version 5.1.0. Red circle: high risk of bias; green circle: low risk of bias; blank: unclear risk of bias.

Estimation of deviation based on Cochrane risk, all trials made mention of a randomized distribution of participants while 36 trials [30, 32–37, 39, 42, 45–50, 58, 59, 61–63, 65, 66, 68, 70–72, 74–77, 79–82, 85, 87] described the appropriate generation of the random allocation sequence. 43 trials [24–26, 32–36, 41, 43–49, 55, 58, 59, 61–67, 69–71, 73–82, 85–87] described the allocation concealment. 34 experiments [23–26, 32–34, 40, 41, 43, 44, 46–49, 55, 59, 61, 62, 65, 66, 71–75, 77–82, 85, 87] mentioned the blindness of the participants, and seven trials [37, 39, 45, 50, 69, 76] referred to the outcome assessment. Sixty-seven studies obtained complete data and were therefore at risk of low loss bias. The risk of reporting deviations was lower in 16 trials [24, 29, 35, 38, 45, 48, 53, 56, 66, 73, 74, 76–78, 80, 88] because of the results of the detailed index reported (Figure 3).

#### 3.1.1. Outcome Measures with Subgroup Analysis: TER of TCMs, ACU, and OTTCM Treatment

**Figure 4:**
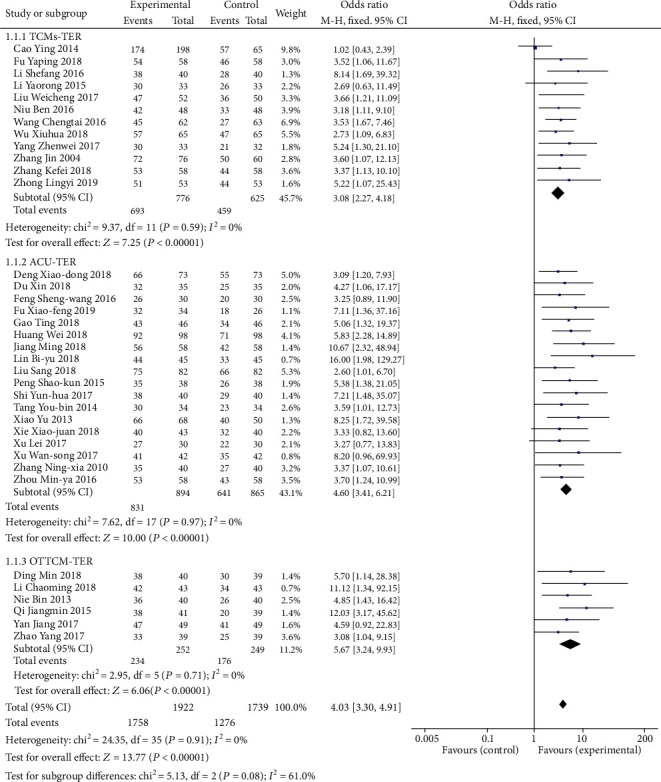
Forest plot of TER treated with TCMs, ACU, and OTTCM alone.

The standard settings for TER are as follows: The symptoms and signs of the patients disappeared, which was defined as a recovery. Apparent effect was identified that the symptoms and signs of the patients were significantly improved. Effectiveness was identified that the symptoms and signs of the patients were improved. The symptoms and signs of the patients were not improved, or even aggravation was defined as invalidation. TER refers to the proportion of patients who were assessed to recovery, the obvious effect, and the effectiveness of total groups. TER was reported in 36 studies. In the TCM group, 12 trials [22, 34, 40–42, 47, 57, 65, 70, 81, 82, 87] mentioned the TER. The results of the meta-analysis of these tests by using a fixed-effects model showed that the combination of TCMs and routine treatment could crucially ameliorate the TER in the treatment of stroke (OR = 3.08, 95% CI: 2.27, 4.18, *P* < 0.00001). There was no statistically significant heterogeneity among single trials (*P* = 0.59, *I*^2^ = 0%). 18 studies [24, 29, 35, 38, 45, 48, 53, 56, 63, 66, 73, 74, 76–80, 88] in the ACU group reported the TER. After a meta-analysis of these trials by using a fixed-effects model, the results depicted that ACU in combination with routine therapy vitally enhanced TER in stroke treatment (OR = 4.60, 95% CI: 3.41, 6.21, *P* < 0.00001). There was no statistically substantial heterogeneity between individual experiments (*P* = 0.97, *I*^2^ = 0%). Six studies [27, 37, 52, 60, 67, 72] in the OTTCM group reported the TER. The results of a fixed-effects model analysis showed that the combination of OTTCMs and routine therapy could significantly improve TER (OR = 5.67, 95% CI: 3.24, 9.93, *P* < 0.00001). There was no statistically remarkable heterogeneity included in individual trials (*P* = 0.71, *I*^2^ = 0%) (Figure 4).

#### 3.1.2. Indices of Blood Lipid of TCMs Combined with Routine Treatment vs. Routine Treatment Alone

**Figure 5:**
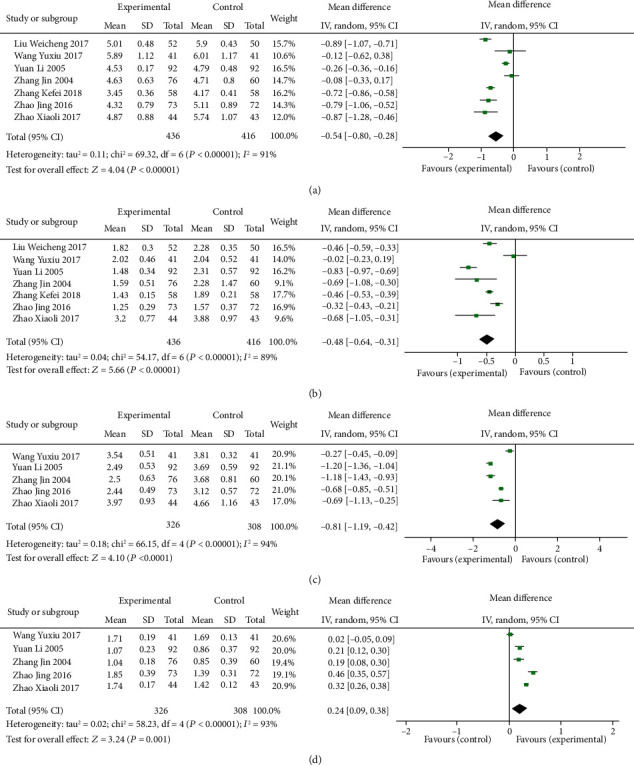
Forest plot of indices of blood lipid in patients treated with TCMs and routine treatment. (a) The plot of TC, (b) the plot of TG, (c) the plot of LDL, and (d) the plot of HDL.

TC, TG, HDL, and LDL were the main indices that mentioned included studies reflected blood lipid. Seven studies [22, 23, 44, 57, 59, 64, 70] reported the detection of TC. Statistical heterogeneity exists between individual studies (*P* < 0.00001, *I*^2^ = 91%), so a random-effects model was applied to take a meta-analysis which demonstrated that the combination of TCMs and routine treatment significantly decreased the level of TC in blood lipid (MD = −0.54, 95% CI: −0.80, −0.28, *P* < 0.0001, Figure 5(a)). Seven trials [22, 23, 44, 57, 59, 64, 70] provided the contents of TG. There was statistically significant heterogeneity among individual studies (*P* < 0.00001, *I*^2^ = 89%), so a random-effects model was applied to take a meta-analysis which demonstrated that the combination of TCMs and routine treatment significantly decreased the level of TG in blood lipids (MD = −0.48, 95% CI: −0.64, −0.31, *P* < 0.00001, Figure 5(b)). Detection of LDL was reported in five trials [22, 23, 44, 59, 64]. Heterogeneity was found among individual studies (*P* < 0.00001, *I*^2^ = 94%), and then, a random-effects analysis was applied to demonstrate that TCMs and routine treatment significantly decreased the level of LDL in blood lipid (MD = −0.81, 95% CI: −1.19, −0.42, *P* < 0.0001, Figure 5(c)). Five studies [22, 23, 44, 59, 64] provided data of HDL. There was heterogeneity among individual trials (*P* < 0.00001, *I*^2^ = 93%) and a meta-analysis using a random-effects analysis proved that combination of TCMs and routine treatment significantly increased the level of HDL in blood lipid (MD = 0.24, 95% CI: 0.09, 0.38, *P* = 0.001, Figure 5(d)).

#### 3.1.3. Indices of hs-CRP, FIB, and HCY of TCMs Combined with Routine Treatment vs. Routine Treatment Alone

**Figure 6:**
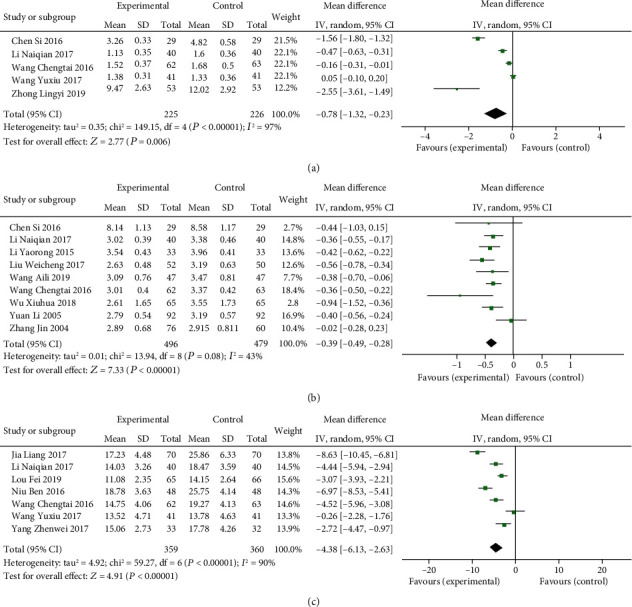
Forest plot of indices of hs-CRP, FIB, and HCY function in patients treated with TCMs and routine treatment. (a) The plot of hs-CRP, (b) the plot of FIB, and (c) the plot of HCY.

Five studies [42, 49, 55, 64, 87] reported the detection of hs-CRP. There was statistically significant heterogeneity among individual studies (*P* < 0.00001, *I*^2^ = 97%), so a random-effects model was applied to take a meta-analysis which demonstrated that the combination of TCMs and routine treatment significantly decreased the level of hs-CRP (MD = −0.78, 95% CI: −1.32, −0.23, *P* = 0.006, Figure 6(a)). Nine trials [22, 23, 40, 42, 49, 55, 57, 81, 84] provided the contents of FIB. There was statistically significant heterogeneity among individual studies (*P* = 0.08, *I*^2^ = 43%), so a random-effects model was applied to take a meta-analysis which demonstrated that the combination of TCMs and routine treatment significantly decreased the level of FIB (MD = −0.39, 95% CI: −0.49, −0.28, *P* < 0.00001, Figure 6(b)). Detection of HCY was reported in seven trials [41, 42, 54, 55, 64, 65, 85]. Heterogeneity in individual researches (*P* < 0.00001, *I*^2^ = 90%) and then a random-effects analysis was applied to demonstrate that TCMs and routine treatment significantly decreased the level of HCY (MD = −4.38, 95% CI: −6.13, −2.63, *P* < 0.00001, Figure 6(c)).

#### 3.1.4. Indices of the National Institutes of Health Stroke Scale of TCMs, ACU, or OTTCM Combined with Routine Treatment vs. Routine Treatment Alone

**Figure 7:**
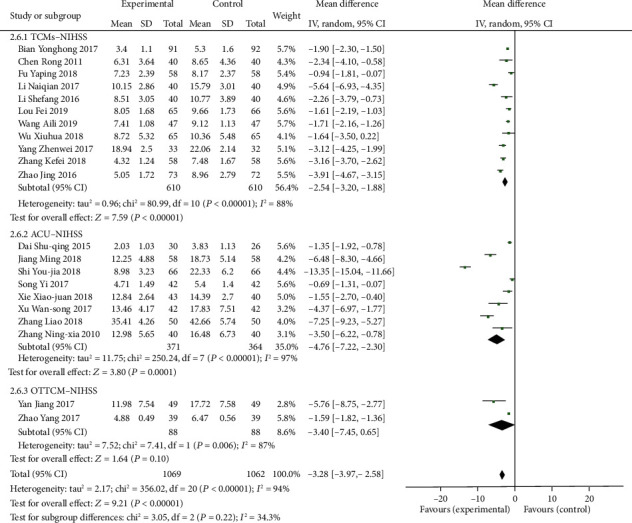
Forest plot of the NIHSS in patients treated with TCMs, ACU, or OTTCM combined with routine treatment.

11 trials [25, 44, 47, 55, 62, 65, 70, 81, 82, 84, 85] in the TCMs group mentioned the NIHSS. A random-effects model was used because of heterogeneity existence (*P* < 0.00001, *I*^2^ = 88%). From the results of the meta-analysis, we can know that TCMs combined with routine treatment can significantly diminish the NIHSS score (MD = −2.54, 95% CI: −3.20, −1.88, *P* < 0.00001). Eight studies [24, 39, 56, 61, 71, 73, 79, 83] reported the NIHSS in the ACU group. A random-effects model was used because of heterogeneity existence (*P* < 0.00001, *I*^2^ = 98%). A meta-analysis showed that ACU combined with routine treatment significantly reduced the NIHSS score (MD = −4.93, 95% CI: −7.58, −2.28, *P* = 0.0003). Two studies [52, 60] reported the NIHSS in the OTTCM group. Due to the existence of heterogeneity, the random-effects model is adopted (*P* = 0.006, *I*^2^ = 87%). A meta-analysis illustrated that the combination of OTCM and routine therapy could greatly lessen the NIHSS score (MD = −3.40, 95% CI: −7.45, 0.65, *P* = 0.10, Figure 7).

#### 3.1.5. Indices of BI of TCMs, ACU, or OTTCM Combined with Routine Treatment vs. Routine Treatment Alone

**Figure 8:**
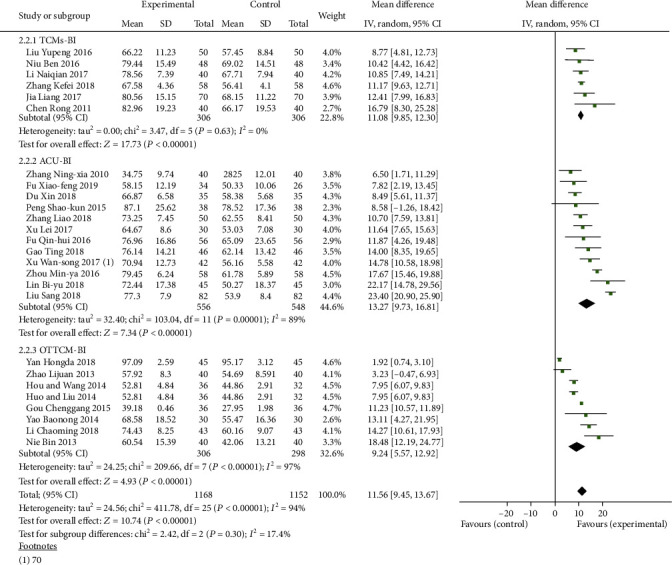
Forest plot of the BI treated with TCMs, ACU, and OTTCM alone.

The BI was mentioned in 6 tests [25, 41, 51, 54, 55, 70] in the TCM group. There was no statistically significant heterogeneity among individual trials (*P* = 0.63, *I*^2^ = 0%). A meta-analysis demonstrated that TCMs combined with routine treatment significantly improved the BI score (MD = 11.08, 95% CI: 9.85, 12.30, *P* < 0.00001). 12 studies [24, 38, 45, 46, 53, 56, 66, 71, 74, 76, 80, 88] reported the BI in the ACU group. A random-effects model was used because of heterogeneity existence (*P* < 0.00001, *I*^2^ = 89%). A meta-analysis showed that ACU combined with routine treatment significantly improved the BI score (MD = 13.27, 95% CI: 9.73, 16.81, *P* < 0.00001). Eight studies [27, 28, 30, 32, 33, 36, 67, 69] reported the BI in the OTTCM group. A random-effects model was used because of heterogeneity existence (*P* < 0.00001, *I*^2^ = 97%). A meta-analysis demonstrated that OTTCM combined with routine treatment significantly decreased the BI (MD = 9.24, 95% CI: 5.57, 12.92, *P* < 0.00001, Figure 8).

#### 3.1.6. Indices of FMA of ACU or OTTCM Combined with Routine Treatment vs. Routine Treatment Alone

**Figure 9:**
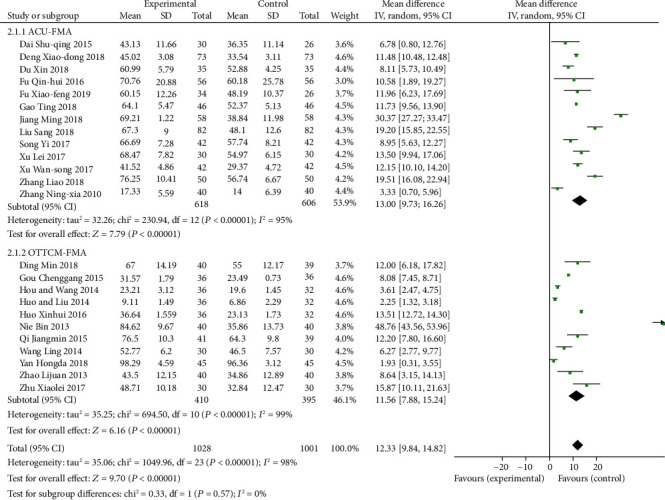
Forest plot of FMA in patients treated with ACU or OTTCM combined with routine treatment.

13 studies [24, 39, 46, 53, 56, 61, 71, 73, 74, 76, 78, 80, 88] reported the FMA in the ACU group. A random-effects model was used because of heterogeneity existence (*P* < 0.00001, *I*^2^ = 95%). A meta-analysis showed that ACU combined with routine treatment significantly improved the FMA score (MD = 13.00, 95% CI: 9.73, 16.26, *P* < 0.00001). 11 studies [27, 28, 31–33, 36, 37, 50, 58, 69, 72] reported the FMA in the OTTCM group. A random-effects model was used because of heterogeneity existence (*P* < 0.00001, *I*^2^ = 99%). The consequences exhibited that OTTCM combined with routine treatment could significantly meliorate the FMA score (MD = 11.56, 95% CI: 7.88, 15.24, *P* < 0.00001, Figure 9).

#### 3.1.7. Hemorheological Indices of TCMs Combined with Routine Treatment vs. Routine Treatment Alone

Hemorheological indices were reported in eligible studies including WHV, WLV, PV, and HCT. Two trials [57, 70] mentioned the WHV and PV level. The MD with 95% CI for WHV and PV were (MD = −0.89, 95% CI: −1.04, −0.74) and (MD = −0.49, 95% CI: −0.68, −0.31), respectively, indicating a significant decrease in the hemorheological indices in the experimental group compared with the control group (*P* < 0.00001). Two trials [57, 70] mentioned the investigation on WLV. The MD with 95% CI for WLV was (MD = −2.30, 95% CI: −4.24, −0.36) certifying a significant increase in the TCMs+routine treatment compared with routine treatment alone (*P* = 0.02). Three trials [40, 59, 62] mentioned the investigation on HCT. The MD with 95% CI for HCT was (MD = −2.65, 95% CI: −4.71, −0.58) certifying a significant increase in the TCMs and routine treatment compared with routine treatment alone (*P* = 0.01, Table 6).

#### 3.1.8. Serum Immunoglobulin of ACU Combined with Routine Treatment vs. Routine Treatment Alone

Serum immunoglobulin was reported in eligible studies including IgA, IgG, and IgM. The serum levels of IgA, IgG, and IgM were measured in one study [75]. The MD with 95% CI for IgA, IgG, and IgM were (MD = −0.77, 95% CI: −1.09, −0.45), (MD = −1.87, 95% CI: −2.51, −1.23), and (MD = −0.91, 95% CI: −1.23, −0.59), respectively, indicating a significant decrease in the serum immunoglobulin in the experimental group compared with the control group (*P* < 0.00001, Table 7).

#### 3.1.9. Observation Index of OTTCM Combined with Routine Treatment vs. Routine Treatment Alone

One study [72] reported the CSI, one trial [69] provided MOCA, and two trials [43, 67] recorded MRS. The MD with 95% CI for CSI was (MD = −1.26, 95% CI: −1.95, −0.57), indicating a significant decrease of CSI in the experimental group (*P* = 0.0004). The MD with 95% CI for MOCA was (MD = 3.39, 95% CI: 1.04, 5.74), indicating a significant increase of MOCA in the experimental group (*P* = 0.005). The MD with 95% CI for MRS was (MD = −0.61, 95% CI: −0.81, −0.42), indicating a significant decrease of MRS in the experimental group (*P* < 0.00001, Table 8).

#### 3.1.10. Swallowing Function Score of ACU Combined with Routine Treatment vs. Routine Treatment Alone

Swallowing function evaluation including SSA and VFSS. One study [68] reported the SSA; two trials [48, 68] provided VFSS. The MD with 95% CI for SSA was (MD = −3.40, 95% CI: −4.99, −1.81), indicating a significant decrease of SSA in the experimental group (*P* < 0.00001). The MD with 95% CI for VFSS was (MD = 2.44, 95% CI: 1.74, 3.14), indicating a significant increase of VFSS in the experimental group (*P* < 0.00001). Five trials [26, 35, 73, 83, 86] provided ADL. The MD with 95% CI for ADL was (MD = 14.04, 95% CI: 7.23, 20.86), indicating a significant increase of ADL in the experimental group (*P* < 0.00001, Table 9).

#### 3.1.11. BFGF and VEGF Expression Levels of ACU Combined with Routine Treatment vs. Routine Treatment Alone

One study [76] reported the BFGF; one trial [76] provided VEGF. The MD with 95% CI for BFGF and VEGF were (MD = 3.90, 95% CI: 2.86, 4.94) and (MD = 272.24, 95% CI: 261.12, 283.36), respectively, indicating a significant increase in the experimental group (*P* < 0.00001, Table 10).

### 3.2. Analysis Diagram of TCM-Index Network Relationship

**Figure 10:**
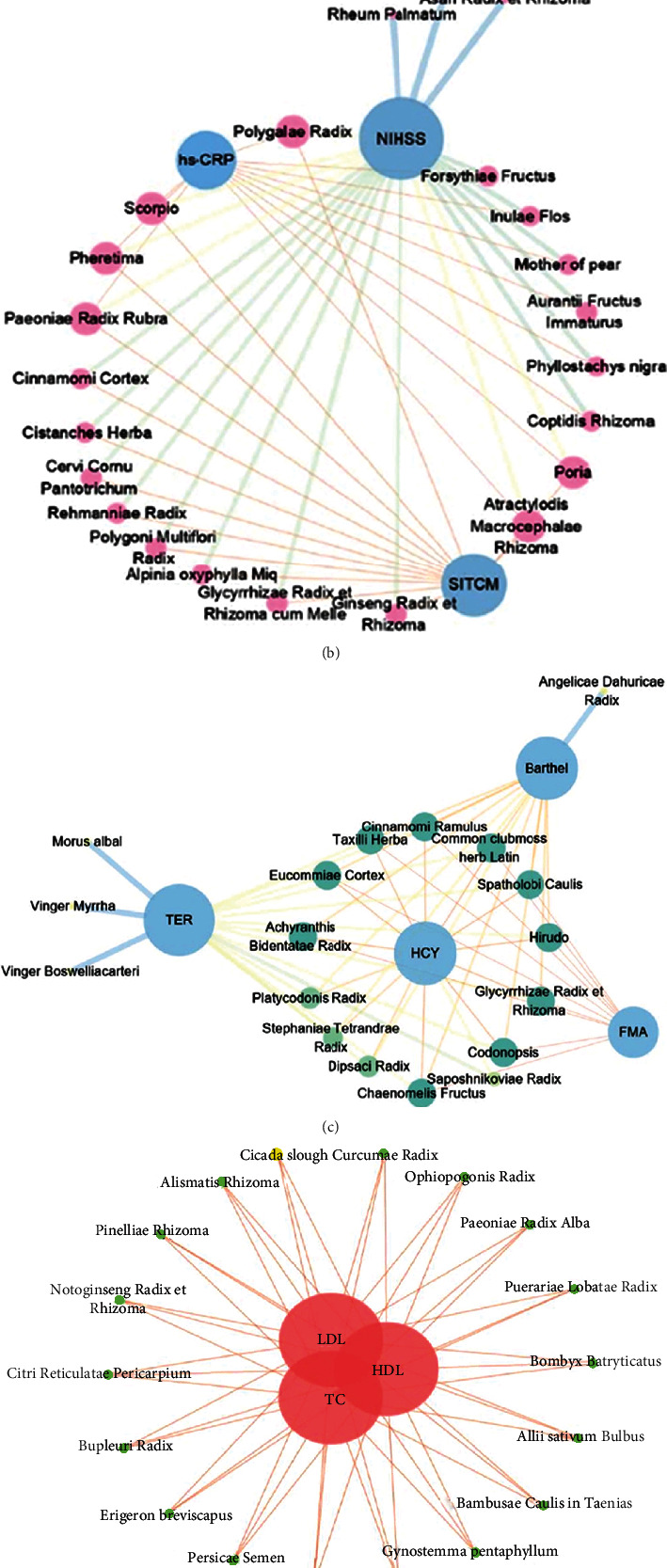
Analysis diagram of TCM-index network relationship.

72 Chinese herbs and 18 related indexes were imported into the Cytoscape3.7.1 software to draw the network analysis map as shown in Figure 10(a). Through the ClusterViz plug-in Cytoscape, four core modules are obtained by using the EAGLE algorithm, as shown in Figures 10(b)–10(e). It can be obtained from Figure 10(a) that TCMs have an obvious recovery effect on all indexes of the apoplexy recovery period. As can be seen from Figure 10(b), the NIHSS, SITCM, and hs-CRP are important indicators of stroke recovery improvement. Rheum Palmatum, Asari Radix et Rhizoma, etc., have an obvious effect on the NIHSS. Pheretima and Achyranthis Bidentatae Radix can enhance the SITCM.  Figure 10(c) can be obtained, common clubmoss herb Latin, Spatholobi Caulis, Glycyrrhizae Radix et Rhizoma, Achyranthes bidentata, etc., can significantly improve the index of TER, HCY, FMA, and BI. As can be obtained in Figure 10(d), Alismatis Rhizoma, Lycii Fructus, and Puerariae Lobatae Radix can improve the level of blood lipids in convalescent patients with stroke. As can be obtained in Figure 10(e), Salviae Mihiorrhizae Radix et Rhizoma, Chuanxiong Rhizoma, and Carthami Flos can improve the indexes of hemorheology (WLV, WHV, PV, and HCT) and reduce the levels of TC and FIB in convalescent patients with stroke (Figure 10)

### 3.3. Analysis Diagram of Acupoint-Index Network Relationship

**Figure 11:**
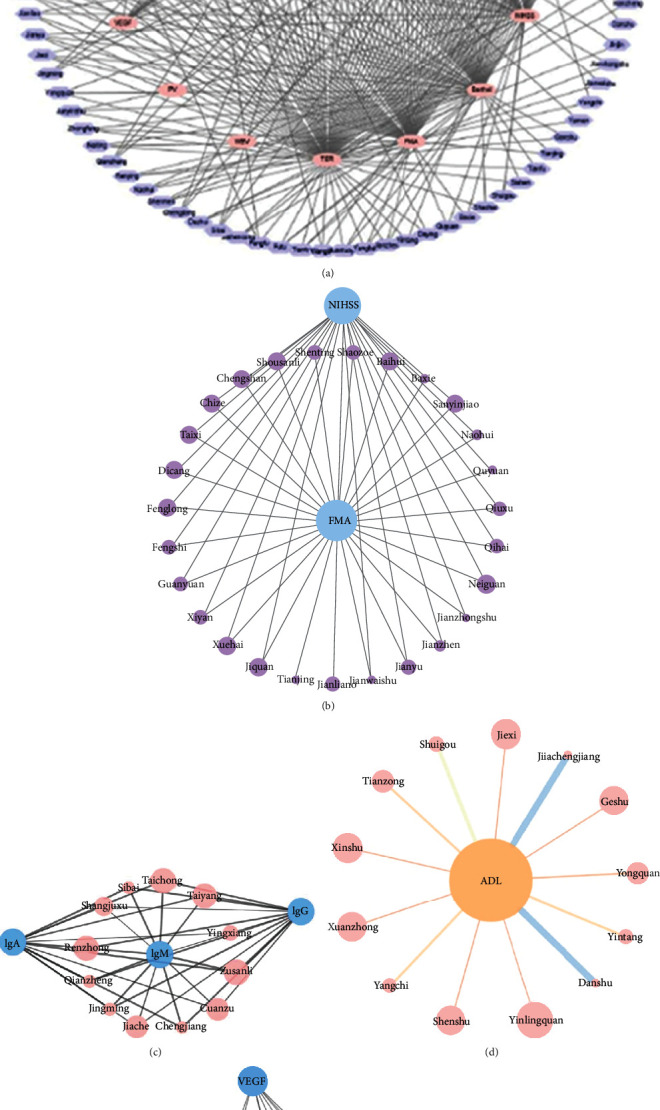
Analysis diagram of ACU-index network relationship.

The 95 acupoints and 16 related indexes were imported into the Cytoscape 3.7.1 software to draw the network analysis diagram in Figure 11(a) through the Cluster Viz plug-in Cytoscape; four core modules were obtained by using the EAGLE algorithm, see Figures 11(b)–11(e). According to Figure 11(a), the following acupoints have obvious effects on the indexes of the stroke recovery stage. As can be seen from Figure 11(b), the NIHSS and FMA are important indicators of stroke recovery improvement. As can be obtained in Figure 11(c), Zusanli (ST36), Renzhong (GV26), Taiyang (EX-HN5), and other acupoints have a significant effect on the serum immunoglobulin index (IgG, IgA, and IgM).  Figure 11(d) shows that YinLingquan (SP9), XuanZhong (GB39), Shenshu (BL23), and other acupoints can significantly improve the score of ADL.  Figure 11(e) shows that Quchi (LI11), Kunlun (BL60), Juegu, and other acupoints have an obvious effect on BFGF. Yanglingquan (SP9), Weizhong (BL40), and Waiguan (TE5) have a significant effect on VEGF. Quchi (LI11), Kunlun (BL60), and Hegu (LI4) have a good effect on improving the BI (Figure 11).

### 3.4. Analysis Diagram of OTTCM-Index Network Relationship

**Figure 12:**
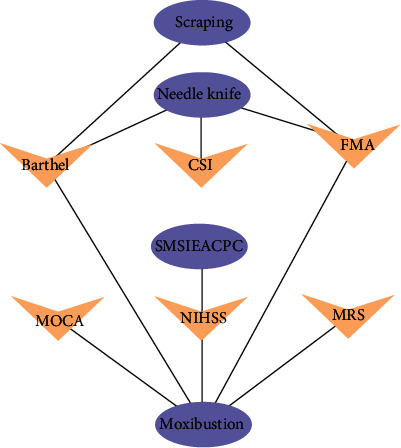
Analysis diagram of OTTCM-index network relationship.

Four kinds of other TCM treatment methods and 8 related indexes are imported into the Cytoscape 3.7.1 software to draw the network analysis map as shown in Figure 12. From the picture, we can see that moxibustion, needle knife, scraping, and internal and external application combined with cupping and other TCM therapy have a significant effect on FMA, CSI, NIHSS, and other indicators (Figure 12).

### 3.5. Publication Bias

**Figure 13:**
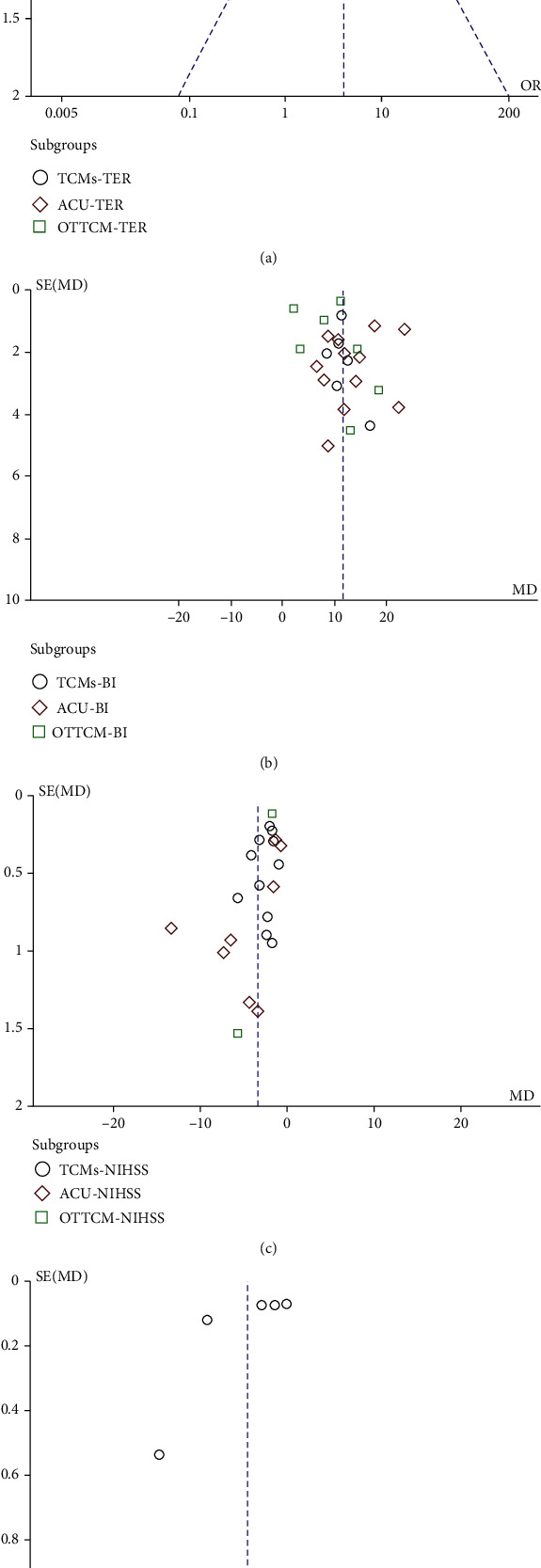
Funnel plot for the publication bias. (a) The plot of TER, (b) the plot of BI, (c) the plot of NIHSS, and (d) the plot of hs-CRP. The funnel plots of these factors were generally symmetrical, indicating that there is no obvious publication bias.

In this study, funnel plots are used to represent publication bias. In this study, funnel plots of a combination of TCM treatment and routine treatment vs. routine treatment alone on NIHSS, BI, TER, and hs-CRP were applied. The plot is generally symmetrical, indicating that there is no obvious publication bias (Figure 13). Egger's test was further executed to examine the publication bias by meta for a package in R platform. We can find that the total NIHSS has publication bias. This is because the OTTCM group only included two articles, so that the Egger's test could not be performed. The hs-CRP indicator also has a publication bias, which may be caused by the small number of documents included (Table 11).

## 4. Discussion

Stroke originated from “Huangdi Neijing,” which is the name of traditional Chinese medicine (TCM). Its clinical manifestations are suddenly faint, hemiplegia, sluggish speech, and tongue skew. It is characterized by acute illness and rapid change, just like the wind [89]. An updated definition of stroke is an acute episode of focal dysfunction of the brain, retina, or spinal cord lasting longer than 24 h. The traditional definition of stroke is clinical and based on the sudden onset of loss of focal neurological function due to infarction or hemorrhage in the relevant part of the brain, retina, or spinal cord [3]. Stroke in the World Health Organization (WHO) is defined as an interruption of blood supply to the brain, usually due to rupture of blood vessels or occlusion of blood clots. Through a large number of reports and authoritative statistical data, it is confirmed that China has become a high-level country of cerebrovascular diseases. Stroke is not only valued in China but also one of the diseases that have aroused great attention in the world [89]. Stroke is also one of the major causes of death worldwide, with about 5.5 million people dying from it every year. The sequelae of stroke also have a significant impact on the quality of life and financial burden of patients and their families. It is estimated that there are about 44 million disability-adjusted life years for stroke survivors, which is the main cause of long-term disability and consumes huge socioeconomic and medical resources [90]. However, long-term disabilities and high recurrence rates remain a cause for concern and pending, prompting patients and their families to seek assistance in complementary therapy [90].

In China, stroke is treated using TCM, which has been developed over thousands of years [18]. The treatment of TCM mainly includes natural medicine, ACU, and physiotherapy. Natural medicine is not only an undeveloped biological resource but also the origin of many new drugs. Among human beings, TCM has a history of more than 2000 years. The precious experience provided by this practice can offer powerful leads for drug discovery [91]. ACU has been proven to lower the risk of stroke recurrence and might be beneficial for muscle spasticity, joint pain, and dysphagia after stroke [90]. At present, the curative effect of western medicine alone in convalescent patients with cerebral infarction is not ideal, and in recent years, a number of studies have confirmed that the TCM treatment including medication, ACU, and physiotherapy has achieved some results.

In recent years, clinical reports have shown that there is a significant correlation between dyslipidemia and the occurrence and development of cerebrovascular disease [64]. Abnormal metabolism of blood lipids, such as elevated TG, TC, and LDL, will increase platelet adhesion, facilitate platelet aggregation, lead to blood coagulation, and lead to vascular endothelial damage, vascular sclerosis, and increased vascular resistance, thereby boosting the development of atherosclerosis. Finally, the degree of hypoxia and ischemia of brain tissue was aggravated. Plasma LDL concentration is a risk factor for ischemic stroke [92]. Therefore, the improvement of hemorheology and blood lipid indexes is of great significance for the treatment of cerebral infarction. Here, we confirm that TCMs protect blood vessels by reducing the content of TC (*P* < 0.0001), TG (*P* < 0.00001), and LDL (*P* < 0.0001), increasing the levels of HDL (*P* = 0.001).

The study found that low hs-CRP (high-sensitivity C-reactive protein) appeared to be associated with a reduced risk of accidental stroke [93]. Based on this, the measurement of hs-CRP has been recommended as a marker of low-grade vessel inflammation in patients at high risk for atherosclerosis in several major guidelines for primary stroke prevention [94]. Fibrinogen (FIB) is an important coagulation factor that plays an important role in regulating thrombosis [95]. FIB is a crucial coagulation factor, which c1an form a reticular structure in plasma. It is an important factor in plasma viscosity and an independent risk factor for cerebral arteriosclerosis. Epidemiological evidence and Mendelian randomization studies indicate that high homocysteine concentrations in the blood are a risk factor for stroke [96]. Here, we found that the medications from TCMs could not only decrease the serum level of hs-CRP (*P* = 0.006) and FIB (*P* < 0.00001) but also decrease HCY (*P* < 0.00001).

The National Institutes of Health Stroke Scale (NIHSS) and Barthel Index (BI) are widely applied scales in stroke research [97]. The NIHSS is an effective and repeatable scale for measuring neurological deficits and is the most commonly used scoring system in stroke intervention trials. The NIHSS reacted to the infarct size, clinical severity, and long-term outcome [98]. The Barthel, originally described in 1955 by Dr. Florence Mahoney and Dorothea Barthel, is a 10-item measure of activities of daily living. Barthel is also a frequently used functional outcome measure for clinical stroke trials, second only to the modified Rankin scale (MRS) in prevalence [99]. In stroke medicine, Barthel is issued in clinical practice to assess baseline abilities and to quantify functional change after rehabilitation. Barthel quantifies ADL in an ordinal, hierarchical scale that ranges from 0 to 20 or 0 to 100 depending on the scoring used [100]. We provided that TCM treatment not only decreased the NIHSS score (*P* < 0.00001) but also increased the Barthel Index (*P* < 0.00001).

The FMA (Fugl-Meyer Assessment) was designed by Fugl-Meyer et al. to provide a numeric score of motor status after stroke based on the sequential stages of motor recovery described by Twitchell, Reynolds et al., and Brunnstrom using measures such as limb synergy and range of motion [101]. FMA is considered by many people in the field of stroke rehabilitation to be one of the most comprehensive quantitative measures of poststroke dyskinesia and has been recommended for clinical trials of stroke rehabilitation [102]. Here, we found that OTTCM and ACU could significantly increase FMA scoring (*P* < 0.00001).

Patients with cerebral infarction usually have a variety of abnormal hemorheological indexes, and the blood often shows a state of high aggregation, resulting in an insufficient supply of oxygen and blood to the local tissue of the brain, resulting in local cerebral necrosis [70]. The results of this study showed that the levels of WHV (*P* < 0.00001), WLV (*P* = 0.02), PV(*P* < 0.00001), and HCT (*P* = 0.01) in the experimental group after treatment were critically lower than those in the control group, indicating that the medications from TCMs can reduce the three levels and improve the abnormal hemorheology of patients.

Immunity is a key factor in the pathobiology of stroke [103]. In the state of abnormal immunity, the abnormal increase of serum IgA, IgG, and IgM levels will lead to or aggravate the inflammatory reaction in the convalescent stage of stroke and further aggravate the severity of stroke. In this study, the levels of serum IgA (*P* < 0.00001), IgG (*P* < 0.00001), and IgM (*P* < 0.00001) in the experimental group were tremendously lower than those in the control group. This result indicates that ACU may contribute to the regulation of immune function in the recovery phase after stroke, although its possible impact is not great. But we can pay more attention to other indicators later.

The modified Rankin score (MRS) was assigned retrospectively by a board-certified neurologist using all the information available. The MRS is a commonly used outcome classification system for indicating the level of disability after cerebral stroke [104]. Mild cognitive impairment in poststroke convalescence is usually screened by the Montreal Cognitive Assessment (MOCA) [105]. The clinical spasticity index (CSI) is a brief, easily administered instrument that is developed for use in preventive clinical practice to identify the strain of informal care providers. The CSI has been applied in many studies to assess the impact of nursing on stroke patients [106]. The MRS (*P* < 0.00001), MOCA (*P* < 0.005), and CSI (*P* < 0.0004) of stroke convalescent patients were meliorated in varying degrees compared to the control group. It shows that OTTCM has a good clinical effect in the treatment of convalescent state after stroke and improves the ability of daily life of patients, so it is worthy of clinical application.

The videofluoroscopy swallow study (VFSS) is considered the gold standard for the detection of swallowing dysfunction [107]. The higher the score, the better the swallowing function. A standardized swallowing assessment (SSA) identified dysphagia and then severity (between 15 and 60 days after stroke) were rated at the time of participation using the VFSS [108]. The higher the score, the worse the swallowing function. In this study, the scores of SSA (*P* < 0.0001) and VFSS (*P* < 0.00001) in the experimental group were vitally improved compared with those in the control group. These results suggest that ACU is helpful to improve the swallowing function of convalescent patients after stroke.

BFGF and VEGF are neurotrophic factors and vasoactive peptides, which can directly repair injured nerve tissue, induce a large number of neovascularizations, enhance microcirculation, lighten brain edema, and play a neuroprotective role [76]. Recent evidence has revealed an important role for vascular endothelial growth factor (VEGF) as a neurotrophic factor and neuroprotectant [109]. In this study, the levels of BFGF (*P* < 0.00001) and VEGF (*P* < 0.00001) in the experimental group were significantly higher than those in the control group. These results suggest that ACU is helpful to enhance the neuroregulatory function of convalescent patients after apoplexy.

It is noteworthy that NIHSS, FMA, TC, TG, LDL, HDL, hs-CRP, HCT, and other indicators have high heterogeneity. In fact, there are many factors that affect heterogeneity, such as the quality of the included literature, gender, age, and geographic location. For instance, in the analysis of the NIHSS index, we can see that the proportion of females in Bian Yonghong 2017 is 46.24% but in Chen Rong2015 is 27.5%. In the analysis of the FMA index, Jiang Ming 2018's geographic location is in Shaanxi Province, but Fu Xiao-feng 2019 is in Zhejiang Province. These are all factors that lead to high heterogeneity of indicators.

The analysis of the relationship between drug and index network shows that Paeoniae Radix Rubra, Angeticae Sinensis Radix, Astragali Radix, Pheretima, Carthami Flos, and Persicae Semen were the most common Chinese medicinal materials. These TCMs are also the prescription composition of Buyang Huanwu decoction (BYHWD), a famous traditional Chinese medicine, which has been utilized to promote the recovery of neurological function in intracerebral hemorrhage for centuries [110]. BYHWT was first described in a medicine book named “Yi Lin Gai Cuo” which was published in 1830 [14]. According to the literature of TCM, Buyang Huanwu decoction has the effect of promoting blood circulation and activating energy (qi) flow. It has been widely used in the clinical treatment and prevention of ischemic cardiocerebrovascular disease in China [111]. The “BYHWT” is comprised of seven natural materials: Astragali Radix, Angeticae Sinensis Radix, Paeoniae Radix Rubra, Pheretima, Chuanxiong Rhizoma, Carthami Flos, and Persicae Semen [14]. The principle drug is Astragali Radix; multiuse can replenish the spleen and stomach, removing blood stasis. The minister drug is Angeticae Sinensis Radix, nourishing blood and promoting blood circulation. The assistant drugs are Paeoniae Radix Rubra, Chuanxiong Rhizoma, Pheretima, Persicae Semen, and Carthami Flos. Pheretima has the effect of assisting other drugs to activate collaterals. Paeoniae Radix Rubra, Chuanxiong Rhizoma, Persicae Semen, and Carthami Flos assist Angeticae Sinensis Radix to remove blood stasis [25]. A large number of studies have shown that BYHWD has a good effect in the treatment of acute cerebral infarction, which can improve the hemodynamic indexes and reduce the inflammatory factors in patients [87]. Puerariae Lobatae Radix, Salviae Mihiorrhizae Radix et Rhizoma, Bombyx Batryticatus, and Spatholobi Caulis are also the most common Chinese herbs. The main prescription group of Gegen Huang qi soup is Astragali Radix, Puerariae Lobatae Radix, Bombyx Batryticatus, cicada slough, and so on. Astragali Radix and Puerariae Lobatae Radix are principle drugs, which can invigorate qi and promote blood circulation [22]. Salviae Mihiorrhizae Radix et Rhizoma and Spatholobi Caulis are minister drugs, which can promote blood circulation and remove blood stasis.

ACU, one of the most popular TCM therapies, has been widely used in the clinical management of stroke [112]. In accordance with the WHO, stroke is one of the most recommended diseases to be treated by ACU [113]. The analysis of the relationship between ACU and index network shows that Baihui (GV20), Sanyinjiao (SP6), and Neiguan (PC6) have an obvious effect on the NIHSS. Baihui (GV20) is the meeting point between the Sanyang meridians of the hands and feet and the du meridian, which can be used for ameliorating infarct volume and neurological function score and exerting a potential neuroprotective role in experimental ischemic stroke [114]. Sanyinjiao (SP6) is the acupoint of Zusanyinjiao, which can improve the obstruction of qi and blood [45]. Neiguan (PC6) can reduce heart rate, suggesting a sympathoinhibitory effect [115]. ACU is helpful to regulate the immune function of convalescent patients after stroke. Renzhong (GV26), Zusanli (ST36), Taiyang (EX-HN5), etc., have an obvious effect on serum immunoglobulin. Yanglingquan (GB34), Xuanzhong (GB39), Geshu (BL17), and so on play an important role in the ADL score. Yanglingquan (GB34) can relieve muscle spasm. Xuan Zhong (GB39) is an acupoint near the ankle joint, which can significantly inhibit local arthritis [116]. Geshu (BL17) has the effect of promoting blood circulation and removing blood stasis, dredging collaterals and relieving pain. Quchi, Waiguan, and so on have obvious influence on Barthel. Quchi (LI11) and Waiguan (TE5) can correct the imbalance between yin and yang. Hegu (LI4) and Huantiao (GB30) play an important role in the VEGF and BFGF indexes. Hegu (LI4) is the main treatment for mouth and eye oblique and apoplectic mouth shiver. Huantiao (GB30) can invigorate the spleen and replenish qi [80].

Moxibustion is a traditional Chinese method that makes use of the heat generated by burning herbal preparations containing Artemisia vulgaris (mugwort) to stimulate acupoints [117]. The procedure has been used for thousands of years in ancient Chinese medicine to restore balance following the belief that imbalance, for whatever reason, causes disorders or diseases [118]. The analysis of the relationship between OTTCM and index network shows that moxibustion plays an important role in FMA, MOCA, and NIHSS. Needle knife is a kind of therapeutic tool that combines ACU with a western scalpel. Its therapeutic effects include the stimulating effect of a needle on acupoints and the effect of a scalpel on cutting [58]. Needle knife can obviously improve CSI, Barthel, and other indexes. Scraping is one of the treasures of TCM and is widely used in the clinic. Scraping has the functions of soothing tendons and dredging collaterals, promoting blood circulation and removing blood stasis, improving microcirculation, promoting metabolism, and so on. Scraping can improve FMA and Barthel. Internal and external application combined with cupping has an obvious effect on the NIHSS score.

## 5. Conclusion

These findings indicate that the combination of TCM treatment and routine treatment significantly improves the TER after routine treatment. These effects are mediated by the combined action of several mechanisms. It is likely that the TCMs combined with routine treatment also affect the blood lipid by regulating the contents of TC, TG, LDL, and HDL. The combination could decrease the expression of thrombus regulatory factor and coagulation effect by decreasing the level of hs-CRP, FIB, and HCY in serum. In the present study, the combination of TCM treatment could enhance the protection of neural function and improve the activity of daily life by decreasing the NIHSS scoring while increasing the BI scoring. The ACU or OTTCM combined with routine treatment displays a motor coordination ability by increasing the level of FMA scoring. TCMs combined with routine treatment can reduce the level of hemorheology by decreasing the level of WHV, WLV, PV, and HCT in serum. In this study, the combination of ACU treatment can improve the immune level of patients by reducing IgG, IgA, and IgM. In this study, the OTTCM combined with routine treatment can enhance the cognitive function and improve the spasmodic state by decreasing CSI and MRS and increasing the MOCA score. The ACU combined with routine treatment can improve swallowing function and activity ability by reducing SSA and increasing the level of VFSS and ADL. The ACU combined with routine treatment plays a neuroprotective role by increasing the levels of BFGF and VEGF. Paeoniae Radix Rubra, Angeticae Sinensis Radix, Astragali Radix, Puerariae Lobatae Radix, and Salviae Mihiorrhizae Radix et Rhizoma can effectively improve the clinical symptoms of stroke convalescent patients, promote the recovery of neurological function, and improve the ability of daily life. Acupuncture of Baihui (GV20), Sanyinjiao (SP6), Neiguan (PC6), Renzhong (GV26), Zusanli (ST36), and Taiyang (EX-HN5) can improve the clinical rehabilitation effect of patients and significantly improve the quality of life of patients. Moxibustion, needle knife, scraping, and other TCM therapy can significantly improve the indexes of stroke patients in the recovery period. However, our findings must be handled with care because of the small sample size and low quality of clinic trials cited. Other rigorous and large-scale RCTs are in need to confirm these results.

## Figures and Tables

**Table 1:** Characteristics of included studies.

	Author, year	Cases T/C	Diagnostic standard	Age (years) range, mean		Sex male/female	
TCMs	Wang Yuxiu, 2017	41/41	DCIS	T: 52-73, 59.8	C: 53-73, 59.9	T: 28/13	C: 29/12
	Zhao Xiaoli, 2017	44/43	NE	T: 43-59, 49.77	C: 42-58, 49.71	T: 27/17	C: 27/17
	Chen Si, 2016	29/29	MPDCD	T: 53-78, 61.15	C: 52-77, 62.18	T: 20/9	C: 18/11
	Li Shefang, 2016	40/40	CDECEA	T, 46-73, 65.9	C: 48-76, 66.3	T: 23/17	C: 22/18
	Zhang Kefei, 2018	58/58	MPDCD	T: 40-73, 58.93	C: 41-75, 59.37	T: 35/23	C: 34/24
	Zhao Jing, 2016	75/75	NR	T: 42-75, 60.6	C: 43-76, 58.8	T: 51/24	C: 48/27
	Yang Zhenwei, 2017	33/32	ATCICE	NR	NR	NR	NR
	Liu Yupeng, 2016	50/50	CEDEA & GPTCDCSTCM	T: 58-70, 61.19	C: 58-70, 61.19	T: 27/23	C: 26/24
	Cao Ying, 2014	198/66	ACILIWM & SDSQDBSS	T: 40-70, 63.1	C: 40-70, 63.3	T: 111/87	C: 45/20
	Fu Yaping, 2018	60/60	GDTAISC (2010)	T: 50-78, 62.1	C: 48-76, 60.2	T: 38/22	C: 36/24
	Li Yaorong, 2015	33/33	CDETEA	T: 34-78	C: 33-77	T: 19/14	C: 18/15
	Wu Xiuhua, 2018	65/65	MPDCD	T: 52-75, 62.3	C: 50-73, 61.2	T: 34/31	C: 36/29
	Liu Weicheng, 2017	53/52	NR	T: 35-80, 36.8	C: 36-78, 59.8	T: 29/24	C: 30/22
	Chen Rong, 2011	40/40	CDECEA & MPDCD	T: 55-82, 70.22	C, 52-78, 66.78	T: 28/12	C: 30/10
	Li Naiqian, 2017	40/40	GDTAISC (2010) & GPCRND	T: 46-75, 64.73	C: 48-78, 64.85	T: 23/17	C: 22/18
	Zhong Lingyi, 2019	53/53	GDAISC	T: 47-78, 59.56	C: 45-77, 60.25	T: 30/23	C: 28/25
	Jia Liang, 2017	70/70	MPDCD	T: 53-75, 64.90	C: 50-73, 64.72	T: 45/25	C: 43/27
	Zhang Jin, 2004	76/60	DCWM & DCTCM	T: 40-70, 57.25	C: 42-71, 58.13	T: 49/27	C: 36/24
	Wang Chengtai, 2016	62/63	GDTAISC (2010) & CDECEA	T: 37-80, 61.17	C: 34-81, 62.25	T: 32/30	C: 32/31
	Niu Ben, 2016	48/48	GDTAISC (2010) & DCQBSS	T: 61-73, 64.9	C: 60-72, 65.7	T: 31/17	C: 33/15
	Wang Aili, 2019	47/47	GDTAISC (2014) & CDECEA	T: 44-79, 57.02	C: 43-76, 56.87	T: 25/22	C: 27/20
	Yuan Li, 2005	92/92	DCTCM	T: 40-70, 58.37	C: 42-71, 58.23	T: 54/38	C: 56/36
	Bian Yonghong, 2017	93/93	GPCRND	T: 47-82, 63.1	C: 44-79, 63.8	T: 51/42	C: 49/44
	Lou Fei, 2019	72/72	GCDTCDC (2016) & CPTCM	T: 45-75, 61.26	C: 45-72, 60.85	T: 44/28	C: 40/32

ACU	Cai Jingjing, 2012	60/60	CDECEA & RDCFNCCD	T: 35-75, 64.5	C: 38-73, 64.6	T: 29/31	C: 31/29
	Chen Dan, 2018	30/30	RDCFNCCD	T: 45-80, 64	C: 38-80, 65	T: 19/11	C: 22/8
	Dai Shuqing, 2015	30/26	MPDVKCD (1997) & SSDEA	T: 60-75, 67.1	C: 54-74, 64.9	T: 19/11	C: 16/10
	Deng Xiaodong, 2018	73/73	GSPISTIAC (2014) & CDTEDSTCM	T: 45-75, 62.8	C: 45-78, 63.7	T: 40/33	C: 39/34
	Du Xin, 2018	35/35	NR	T: 41-65, 51.32	C: 39-72, 50.67	T: 18/17	C: 16/19
	Feng Shengwang, 2016	30/30	GDTAISC	T: 48-72, 60	C: 46-71, 58	T: 18/12	C: 19/11
	Fu Qinhui, 2016	56/56	CDECEA	T: 65.68	C: 63.80	T: 36/20	C: 35/21
	Fu Xiaofeng, 2019	34/26	RDCFNCCD & DCAFSATCM	T: 60.35	C: 62.33	T: 18/16	C: 14/12
	Gao Ting, 2018	46/46	GDTAISC	T: 63.76	C: 64.23	T: 25/21	C: 27/19
	Huang Wei, 2018	98/98	GDTAISC	NR	NR	NR	NR
	Jiang Ming, 2018	58/58	RDCFNCCD	T: 58.47	C: 57.02	T: 38/20	C: 39/19
	Lin Biyu, 2018	45/45	NR	T: 47-73, 65.32	C: 46-71, 64.48	T: 32/13	C: 30/15
	Liu Sang, 2018	82/82	MPDVKCD & SSDEA	T: 66.7	C: 67.9	T: 56/26	C: 51/31
	Peng Shaokun, 2015	38/38	SSDEA	T: 43-78, 61.6	C: 41-81, 61.2	T: 22/16	C: 24/14
	Shi Youjia, 2018	66/66	NR	NR	NR	NR	NR
	Shi Yunhua, 2017	40/40	CDECEA	T: 51-78, 68.4	C: 50-78, 68.6	T: 25/15	C: 24/16
	Song Yi, 2017	42/42	SSDEA	T: 60.43	C: 60.29	T: 19/23	C: 24/18
	Tang Youbin, 2014	34/34	SSDEA	T: 31-64, 43	C: 28-66, 38	T: 23/11	C: 28/6
	Wang Jing, 2019	35/35	NR	NR	NR	NR	NR
	Xiao Yu, 2013	68/50	NR	T: 40-71, 51.9	C: 41-72, 52.1	T: 41/27	C: 31/19
	Xie Xiaojuan, 2018	43/40	MPDVKCD	T: 56.94	C: 58.15	T: 22/21	C: 21/19
	Xu Lei, 2017	30/30	CDTEDSTCM	T: 40-74, 58	C: 42-74, 61	T: 15/15	C: 16/14
	Xu Wansong, 2017	42/42	MPDVKCD	T: 44-71, 56.18	C: 45-73, 56.32	T: 28/14	C: 25/17
	Zhang Liao, 2018	50/50	GPTCDC & CDECEA	T: 40-77, 55.9	C: 41-76, 56.4	T: 34/16	C: 33/17
	Zhang Ningxia, 2010	40/40	RDCFNCCD	T: 40-80, 65.9	C: 40-80, 69.2	T: 26/14	C: 24/16
	Zhou Minya, 2016	58/58	MPDVKCD	T: 59.45	C: 58.16	T: 39/19	C: 35/23
	Zhou Shuxin, 2018	60/60	RDCFNCCD	T: 49-84, 69.1	C: 51-85, 70.4	T: 38/22	C: 41/19

OTTCM	Yao Baonong, 2014	30/30	CDECEA (1995)	T: 50-76, 60.9	C: 52-77, 61.7	T: 18/22	C: 16/14
	Zhu Xiaolei, 2017	30/30	GPTCDC (2007)	T: 51-76, 61.5	C: 50-78, 62.3	T: 16/14	C: 17/13
	Yan Jiang, 2017	49/49	NR	T: 55-75, 62.9	C: 55-75, 63.1	T: 32/17	C: 31/18
	Qi Jiangmin, 2017	41/39	MPDAKCD & CDECEA	T: 18-75, 53.6	C: 18-75, 52.8	T: 25/16	C: 17/22
	Huo and Wang, 2014	36/32	CDECEA (1996)	T: 59	C: 62	T: 20/16	C: 18/14
	Guo Chenggang, 2015	36/36	DCCA (1995)	T: 55.7	C: 56.2	T: 20/16	C: 19/17
	Ding Min, 2018	40/39	CDECEA (1996) & MPDAKCD	T: 46-69, 61.0	C: 44-70, 60.2	T: 19/21	C: 20/19
	Yan Hongda, 2018	45/45	NR	T: 65.65	C: 63.21	T: 25/20	C: 28/17
	Zhao Lijuan, 2013	40/40	NR	T: 44-78, 62.72	C: 48-80, 62.56	T: 22/18	C: 19/21
	Nie Bin, 2013	40/40	CETEDA & MPDAKCD	T: 38-75, 58	C: 38-75, 59	T: 23/17	C: 21/19
	Hou and Liu, 2014	36/32	CDECEA (1996)	T: 59.2	C: 61.8	T: 20/16	C: 18/14
	Huo Xinhui, 2016	36/32	MPDAKCD & CDECEA	T: 52.25	C: 54.88	T: 25/11	C: 19/13
	Yang Haixia, 2016	30/30	ESCETCMDA (1995) & GDTALSC (2010)	T: 28-67	C: 31-70	T: 20/10	C: 18/12
	Li Chaoming, 2018	43/43	CDTEDSTCM & DCA	T: 48-76, 62.4	C: 46-77, 63.1	T: 25/18	C: 24/19
	Zhao Yang, 2017	39/39	NR	T: 48-74, 62.04	C: 47-73, 61.75	T: 22/17	C: 21/18
	Wang Ling, 2014	30/30	SDTRMC	T: 53.53	C: 51.53	T: 21/9	C: 18/12

ATCICE: atherosclerotic thrombotic cerebral infarction or cerebral embolism; ACU: acupuncture; ACILIWM: arteriosclerosis cerebral infarction or lacunar infarction in western medicine; C: control group; CEDEA: criteria for evaluation of diagnostic efficacy of apoplexy; CDECEA: criteria for diagnosis and evaluation of curative effect of apoplexy; CDTEDSTCM: criteria for diagnosis and therapeutic effect of diseases and syndromes of traditional Chinese medicine; CETEDA: criteria for evaluation of therapeutic effect in the diagnosis of apoplexy; CPTCM: clinical pathway of traditional Chinese medicine in 22 specialties and 95 diseases; CDETEA: criteria for diagnosis and evaluation of therapeutic effect of apoplexy in traditional Chinese medicine; DCAFSATCM: diagnostic criteria for apoplexy formulated by the state administration of traditional Chinese medicine; DCA: diagnostic criteria for apoplexy; DCCA: diagnostic criteria of cerebral apoplexy; DCIS: diagnostic criteria of ischemic stroke; DCQBSS: diagnostic criteria of qi deficiency and blood stasis syndrome in traditional Chinese medicine; DCTCM: diagnostic criteria of traditional Chinese medicine; DCWM: diagnostic criteria of western medicine; ESCETCMDA: evaluation standard of curative effect of traditional Chinese medicine diagnosis of apoplexy; GSPISTIAC: guidelines for secondary prevention of ischemic stroke and transient ischemic attack in China; GCDTCDC: guidelines and consensus for diagnosis and treatment of cerebrovascular diseases in China; GPTCDC: guidelines for the prevention and treatment of cerebrovascular diseases in China; GDAISC: guidelines for the diagnosis of acute ischemic stroke in China; GDTAISC: guidelines for the diagnosis and treatment of acute ischemic stroke in China; GDTALSC: guidelines for the diagnosis and treatment of acute ischemic stroke in China 2010; GPCRND: guiding principles for clinical research of new drugs of traditional Chinese medicine; GPTCDCSTCM: guidelines for the prevention and treatment of cerebrovascular diseases of the Chinese society of traditional Chinese medicine; MPDVKCD: main points of diagnosis of various kinds of cerebrovascular diseases; MPDCD: main points of diagnosis of all kinds of cerebrovascular diseases; MPDAKCD: main points of diagnosis of all kinds of cerebrovascular diseases; NR: no report; NE: neurology; OTTCM: other treatments of traditional Chinese medicine; RDCFNCCD: reference to the diagnostic criteria of the fourth National Conference on Cerebrovascular Diseases; SDSQDBSS: syndrome differentiation standard of qi deficiency and blood stasis syndrome in traditional Chinese medicine; SDTRMC: standard for diagnosis and treatment of rehabilitation medicine in China; SSDEA: scoring standard for diagnostic efficacy of apoplexy; T: trial group; TCMs: traditional Chinese medicines.

**Table 2:** Intervention characteristics of included studies.

	Study ID (name, year)	Treatment group	Control group	Duration/follow-up	Outcome measures
TCMs	Wang Yuxiu, 2017	TCM-1 (3 tablets, tid)+RT	RT	8 weeks/NR	TC, TG, LDL, HDL, hs-CRP, HCY
	Zhao Xiaoli, 2017	TCM-3, bid+TCM-4, bid+RT	RT	2 months/NR	TC, HDL, LDL, TG, HCT
	Chen Si, 2016	TCM-5, tid+RT	RT	14 days/NR	hs-CRP, FIB
	Li Shefang, 2016	TCM-6, ivdrip, qd+RT	RT	1 month/NR	TER, NIHSS
	Zhang Kefei, 2018	TCM-7, po+RT	RT	4 weeks/NR	TG, TC, NIHSS, Bl, WHV, WLV, PV
	Zhao Jing, 2016	TCM-1 (3 tablets, tid)+RT	RT	12 months/NR	TC, TG, HDL, LDL, NIHSS
	Yang Zhenwei, 2017	TCM-8, po, qd+RT	RT	3 months/NR	NIHSS, HCY
	Liu Yupeng, 2016	TCM-2+RT	RT	4 weeks/NR	FMA, Bl
	Cao Ying, 2014	TCM-9, 4 tablets, tid+RT	RT	28 days/NR	TER
	Fu Yaping, 2018	TCM-10, 200 ml/d, bid+RT	RT	8 weeks/NR	TER, SITCM, NIHSS
	Li Yaorong, 2015	TCM-11, ivdrip, qd+RT	RT	14 days/NR	TER, HCT, FIB
	Wu Xiuhua, 2018	TCM-12, 3 tablets, tid+RT	RT	3 months/NR	TER, NIHSS, FIB
	Chen Rong, 2011	TCM-13, bid+RT	RT	14 days/NR	NIHSS, Bl
	Zhong Lingyi, 2019	TCM-2, bid+RT	RT	8 weeks/NR	TER, hs-CRP
	Jia Liang, 2017	TCM-14, bid+RT	RT	8 weeks/NR	FMA, Bl, HCY
	Zhang Jin, 2004	TCM-15, bid+RT	RT	28 days/NR	TER, FIB, TC, TG, LDL, HDL
	Wang Chengtai, 2016	TCM-16, bid+RT	RT	4 weeks/NR	TER, hs-CRP, FIB, HCY
	Niu Ben, 2016	TCM-17, bid+RT	RT	8 weeks/NR	TER, BI, HCY
	Wang Aili, 2019	TCM-2, bid+RT	RT	6 weeks/NR	NIHSS, SITCM, FIB, TER
	Yuan Li, 2005	TCM-18, 0.4 g/time, ivdrip+RT	RT	28 days/NR	TER, FIB, TC, TG, HDL, LDL
	Liu Weicheng, 2017	TCM-19, tid, 2 bags/time+RT	RT	1 month/NR	TER, TC, TG, FIB, WHV, WLV, PV
	Li Naiqian, 2017	TCM-20, 200 ml/d, bid+RT	RT	15 days/12 months	NIHSS, Bl, HCY, hs-CRP, FIB
	Bian Yonghong, 2017	TCM-21, 200 ml/d, bid+RT	RT	8 weeks/NR	NIHSS, HCT
	Lou Fei, 2019	TCM-22, 3 tablets, tid+RT	RT	12 weeks/NR	NIHSS, HCY, SITCM

ACU	Cai Jingjing, 2012	Acupuncture+RT1	RT1	4 weeks/NR	ADL
	Chen Dan, 2018	Acupuncture+RT	RT	4 weeks/NR	SSA, VFSS
	Dai Shuqing, 2015	Acupuncture, qd+RT2	RT2	36 days/NR	FMA, NIHSS
	Deng Xiaodong, 2018	Acupuncture, qd+RT	RT	3 weeks/NR	TER, FMA
	Du Xin, 2018	Acupuncture, qd+RT3	RT3	4 weeks/NR	TER, FMA, BI
	Feng Shengwang, 2016	Acupuncture, qd+RT4	RT4	3 weeks/NR	TER, VFSS
	Fu Qinhui, 2016	Acupuncture, qd+RT	RT	8 weeks/NR	FMA, BI
	Fu Xiaofeng,2019	Acupuncture, qd+RT	RT	4 weeks/NR	TER, FMA, BI
	Gao Ting, 2018	Acupuncture, qd+RT5	RT5	4 weeks/NR	TER, FMA, BI, BFGF, VEGF
	Huang Wei, 2018	Acupuncture, qd+RT	RT	4 weeks/NR	TER
	Jiang Ming, 2018	Acupuncture, qd+RT6	RT6	4 weeks/NR	TER, FMA, ADL, NIHSS
	Lin Biyu, 2018	Acupuncture, qd+RT7	RT7	20 days/NR	TER, BI
	Liu Sang, 2018	Acupuncture, qd+RT	RT	2 weeks/NR	TER, FMA, BI
	Peng Shaokun, 2015	Acupuncture, qd+RT	RT	3 weeks/NR	TER, BI
	Shi Youjia, 2018	Acupuncture, qd+RT	RT	NR/NR	ADL, NIHSS
	Shi Yunhua, 2017	Acupuncture, qod+RT	RT	4 weeks/NR	TER
	Song Yi, 2017	Acupuncture, qd+RT8	RT8	4 weeks/5 months	FMA, NIHSS
	Tang Youbin, 2014	Acupuncture, qd+RT	RT	NR/NR	TER, ADL
	Wang Jing, 2019	Acupuncture, qd+RT9	RT9	12 weeks/3 months	ADL
	Xiao Yu, 2013	Acupuncture, qd+RT	RT	12 weeks/NR	TER
	Xie Xiaojuan, 2018	Acupuncture, qd+RT	RT	4 weeks/NR	TER, NIHSS
	Xu Lei, 2017	Acupuncture, qd+RT	RT	40 days/NR	TER, FMA, BI
	Xu Wansong, 2017	Acupuncture, qd+RT	RT	40 days/NR	TER, FMA, BI, NIHSS
	Zhang Liao, 2018	Acupuncture, qd+RT10	RT10	10 weeks/NR	FMA, BI, NIHSS
	Zhang Ningxia, 2010	Acupuncture, qd+RT11	RT11	3 weeks/NR	TER, FMA, BI, NIHSS
	Zhou Minya, 2016	Acupuncture, qd+RT	RT	4 weeks/NR	TER, BI
	Zhou Shuxin, 2018	Acupuncture, qd+RT12	RT12	4 weeks/NR	IgA, IgG, IgM

OTTCM	Yao Baonong, 2014	Moxibustion+RT	RT	1 month/NR	Bl
	Zhu Xiaolei, 2017	Needle knife+RT	RT	4 weeks/NR	FMA
	Yan Jiang, 2017	SSIEAC+RT	RT	15 days/NR	NIHSS, TER
	Qi Jiangmin, 2017	Moxibustion+RT	RT	1 week/NR	FMA, TER
	Huo and Wang, 2014	Moxibustion+RT	RT	20 days/1 month	FMA, Barthel
	Guo Chenggang, 2015	Needle knife+RT	RT	6 weeks/NR	FMA, Barthel
	Ding Min, 2018	Needle knife+RT	RT	15 days/NR	FMA, CSI, TER
	Yan Hongda, 2018	Moxibustion+RT	RT	8 weeks/NR	MOCA, Barthel, FMA
	Zhao Lijuan, 2013	SWM+RT	RT	15 days/NR	FMA, Barthel
	Nie Bin, 2013	Moxibustion+RT	RT	2 months/NR	FMA, Barthel, TER
	Hou and Liu, 2014	Moxibustion+RT	RT	20 days/1 month	FMA, Barthel
	Huo Xinhui, 2016	Moxibustion+RT	RT	1 month/1 month	FMA
	Yang Haixia, 2016	Moxibustion+RT	RT	1 month/NR	MRS
	Li Chaoming, 2018	Moxibustion+RT	RT	4 weeks/NR	Barthel, MRS, TER
	Zhao Yang, 2017	Moxibustion+RT	RT	2 months/NR	NIHSS, TER
	Wang Ling, 2014	Moxibustion+RT	RT	3 weeks/NR	FMA

ADL: evaluation result of activities of daily living; bid: twice a day; BI: Barthel Index; BFGF: serum fibrillar growth factor; CSI: clinical spasticity index; FMA: Fugl-Meyer Assessment; FIB: fibrinogen; hs-CRP: hypersensitive C-reactive protein; HDL: high-density lipoprotein; HCY: homocysteine; HCT: hematocrit; ivdrip: intravenous drip; IgA: immunoglobulin A; IgG: Immunoglobulin G; IgM: immunoglobulin M; LDL: low-density lipoprotein; MRS: modified Rankin scale; NIHSS: National Institutes of Health Stroke Scale; po: oral administration; PV: plasma viscosity; qd: once a day; RT: routine treatment; SITCM: syndrome integral of traditional Chinese medicine; SSA: standardized swallowing assessment; SSIEAC: self-made square internal and external application combined with puncture and cupping; SWM: scrapping with moxibustion; TER: total efficacy rate; TC: total cholesterol; TG: triglyceride; tid: three times a day; VEGF: vascular endothelial growth factor; VFSS: videofluoroscopy swallowing study; WHV: whole high viscosity; WLV: whole low viscosity.

**Table 3:** Prescription of TCM and its source.

Drugs	Prescription name	Composition	Prescription	Source
TCM-1	Yindan Xinnao Tong soft capsule	Ginkgo Folium, Salviae Mihiorrhizae Radix et Rhizoma, Erigeron breviscapus, Gynostemma pentaphyllum, Crataegi Fructus, Allii sativum Bulbus, Notoginseng Radix et Rhizoma, L-Borneolum		《Chinese Pharmacopoeia》
TCM-2	Buyang Huanwu decoction	Astragali Radix, Angeticae Sinensis Radix, Paeoniae Radix Rubra, Pheretima, Chuanxiong Rhizoma, Carthami Flos, Persicae Semen		《Yi Lin Gai Cuo》
TCM-3	Added flavor of Buzhong Yiqi decoction	Astragali Radix 30 g, Codonopsis 20 g, (Angeticae Sinensis Radix, Atractylodis Macrocephalae Rhizoma, Alismatis Rhizoma, Bupleuri Radix, Rehmanniae Radix, Puerariae Lobatae Radix, Chuanxiong Rhizoma, Achyranthis Bidentatae Radix) 15 g, (Ophiopogonis Radix, Gastrodiae Rhizoma) 12 g, (Pheretima, Citri Reticulatae Pericarpium, Carthami Flos) 9 g	Buzhongyiqi decoction	《Yi Lin Gai Cuo》
TCM-4	Shenmatongluo capsules	Astragali Radix, Salviae Mihiorrhizae Radix et Rhizoma, Gastrodiae Rhizoma, Pinelliae Rhizoma, Paeoniae Radix Rubra, Angeticae Sinensis Radix, Persicae Semen, Carthami Flos, Spatholobi Caulis, Bambusae Caulis in Taenias, Acori Tatarinowii Rhizoma, Bombyx Batryticatus, Lycii Fructus, Curcumae Radix, Paeoniae Radix Alba, Achyranthis Bidentatae Radix		《Chinese Pharmacopoeia》
TCM-5	Shenqi Fuzheng injection	Codonopsis, Astragali Radix		《Chinese Pharmacopoeia》
TCM-6	Shuxuetong injection	Hirudo, Pheretima		《Chinese Pharmacopoeia》
TCM-7	Pinggan Ditan Tongluo decoction	Gastrodiae Rhizoma 10 g, Uncariae Ramulus cum Uncis 20 g, Salviae Mihiorrhizae Radix et Rhizoma 15 g, Notoginseng Radix et Rhizoma 15 g, Angeticae Sinensis Radix 15 g, Chuanxiong Rhizoma 15 g, Acori Tatarinowii Rhizoma 10 g, Persicae Semen 10 g, Carthami Flos 10 g, Glycyrrhizae Radix et Rhizoma 6 g	Kang Xian Jian	Self-formulation
TCM-8	Quyu Huatan Tongfu recipe	Coptidis Rhizoma9 g, Forsythiae Fructus 9 g, Phyllostachys nigra 9 g, Inulae Flos 9 g, Aurantii Fructus Immaturus 9 g, Puerariae Lobatae Radix 30 g, Hirudo 3 g, fried Crataegi Fructus 10 g, Poria 10 g, Rheum Palmatum 5 g, Notoginseng Radix et Rhizoma 3 g, mother of pear 15 g	Huanglian Wendan decoction	《Liu Yin Tiao Bian》
TCM-9	Naoxintong capsule	Astragali Radix, Paeoniae Radix Rubra, Salviae Mihiorrhizae Radix et Rhizoma, Angeticae Sinensis Radix, Chuanxiong Rhizoma, Persicae Semen, Carthami Flos, Vinger Boswelliacarteri, Vinger Myrrha, Spatholobi Caulis, Achyranthis Bidentatae Radix, Cinnamomi Ramulus, Morus albal, Pheretima, Scorpio, Hirudo		《Chinese Pharmacopoeia》
TCM-10	Yiqi Huoxue Tongluo Tang	Astragalus mongholicus Bunge60 g, Ginseng Radix et Rhizoma 30 g, (Persicae Semen, Carthami Flos, Salviae Mihiorrhizae Radix et Rhizoma, Angeticae Sinensis Radix) 15 g, (Paeoniae Radix Rubra, Chuanxiong Rhizoma, Hirudo, Pheretima, Atractylodis Macrocephalae Rhizoma, Alpinia oxyphylla Miq, Acori Tatarinowii Rhizoma, Polygalae Radix) 12 g, Glycyrrhizae Radix et Rhizoma cum Melle 6 g	Buyang Huanwu decoction	《Yi Lin Gai Cuo》
TCM-11	Shuxuening injection	Ginkgo Folium		《Chinese Pharmacopoeia》
TCM-12	Maixuekang capsule	Hirudo		《Chinese Pharmacopoeia》
TCM-13	Modified Buyang Huanwu decoction	Astragali Radix 60 g, Persicae Semen 10 g, Carthami Flos 10 g, Angeticae Sinensis Radix 10 g, Chuanxiong Rhizoma 10 g, Pheretima 10 g, Erigeron breviscapus 30 g, Hirudo 10 g, Asari Radix et Rhizoma 10 g, Puerariae Lobatae Radix 30 g, Acori Tatarinowii Rhizoma 15 g, Angelicae Dahuricae Radix 8 g	Buyang Huanwu decoction	《Yi Lin Gai Cuo》
TCM-14	Traditional Chinese medicine for tonifying qi and promoting blood circulation	Astragali Radix 30 g, Codonopsis 20 g, Achyranthis Bidentatae Radix 20 g, Pheretima 15 g, Taxilli Herba 15 g, Spatholobi Caulis 15 g, Cinnamomi Ramulus 10 g, Angeticae Sinensis Radix 10 g, Paeoniae Radix Rubra 10 g, Chuanxiong Rhizoma 10 g, Eucommiae Cortex 10 g, common clubmoss herb Latin 10 g, Glycyrrhizae Radix et Rhizoma 10 g	Buyang Huanwu decoction	《Yi Lin Gai Cuo》
TCM-15	Gegen huangqi soup	Astragali Radix 30 g~60 g, Puerariae Lobatae Radix 30 g, Salviae Mihiorrhizae Radix et Rhizoma 12 g, Bombyx Batryticatus 10 g, Spatholobi Caulis 25 g, Angeticae Sinensis Radix 6 g, cicada slough 10 g, Scorpio 5 g	Zhufeng Tongbi decoction	
TCM-16	Qi-tonifying and stasis-eliminating therapy	Astragalus mongholicus Bunge 60 g, (Pheretima, Paeoniae Radix Rubra) 15 g, Angeticae Sinensis Radix 12 g, (Saposhnikoviae Radix, Chuanxiong Rhizoma) 10 g, (Hirudo, Scorpio) 6 g	Buyang Huanwu decoction	《Yi Lin Gai Cuo》
TCM-17	Buqi Jiannao Tongluo Tang	Astragali Radix 60 g, Codonopsis 20 g, Angeticae Sinensis Radix 10 g, Paeoniae Radix Rubra 10 g, Chuanxiong Rhizoma 10 g, Achyranthis Bidentatae Radix 15 g, Pheretima 15 g, Cinnamomi Ramulus 10 g, Dipsaci Radix 15 g, Taxilli Herba 15 g, Eucommiae Cortex 10 g, Chaenomelis Fructus 10 g, common clubmoss herb Latin 10 g, Stephaniae Tetrandrae Radix 15 g, Hirudo 10 g, Spatholobi Caulis 15 g, Platycodonis Radix 10 g, Glycyrrhizae Radix et Rhizoma 10 g	Buyang Huanwu decoction	《Yi Lin Gai Cuo》
TCM-18	Puerarin	Puerariae Lobatae Radix		《Chinese Pharmacopoeia》
TCM-19	Lemai granule	Salviae Mihiorrhizae Radix et Rhizoma, Chuanxiong, Rhizoma, Paeoniae Radix Rubra, Carthami Flos, Cyperi Rhizoma, Aucklandiae Radix, Crataegi Fructus		《Chinese Pharmacopoeia》
TCM-20	Yiqi Huoxue Huatan Tongluo decoction	Astragali Radix 30 g, Paeoniae Radix Rubra 25 g, Angeticae Sinensis Radix 15 g, (Persicae Semen, Chuanxiong Rhizoma, Citri Reticulatae Pericarpium, Bambusae Caulis in Taenias, Pinelliae Rhizoma Praeparatum, Aurantii Fructus Immaturus, Acori Tatarinowii Rhizoma, Polygalae Radix) 10 g, (Carthami Flos, Glycyrrhizae Radix et Rhizoma) 6 g	Buyang Huanwu decoction	《Yi Lin Gai Cuo》
TCM-21	Qingnao Shuluo decoction	Astragali Radix Praeparata Cum Malle 30 g, Angeticae Sinensis Radix 10 g, Paeoniae Radix Rubra 10 g, Persicae Semen 10 g, Carthami Flos 6 g, Pheretima 10 g, Salviae Mihiorrhizae Radix et Rhizoma 15 g, Chuanxiong Rhizoma 10 g, Spatholobi Caulis 30 g, Taxilli Herba 15 g, Achyranthis Bidentatae Radix 15 g, Liquidambaris Fructus 20 g, Crataegi Fructus 15 g	Buyang Huanwu decoction	《Yi Lin Gai Cuo》
TCM-22	Peiyuan Tongzhi capsule	Polygoni Multiflori Radix, Rehmanniae Radix, Cervi Cornu Pantotrichum, Cistanches Herba, Cinnamomi cortex, Scorpio, Pheretima, Hirudo, Paeoniae Radix Rubra, Poria, fried Crataegi Fructus, Glycyrrhizae Radix et Rhizoma		《Chinese Pharmacopoeia》

**Table 4:** Acupoint control table.

Study ID (name, year)	Acupoint
Cai Jing-jing, 2012	Jianliao, Quchi, Hegu, Huantiao, Yanlingquan, Zusanli, Xuanzhong, Jiexi, Kunlun, Taichong, Dicang, Yingxiang, Jiachengjiang, Lianquan
Chen Dan, 2018	Lianquan, Fengchi, Fengfu
Dai Shu-qing, 2015	Guanyuan, Qihai, Zusanli, Jianzhen, Naoshu, Bingfeng, Tianzong, Quyuan, Jianwaishu, Jianzhongshu, Jianyu, Quchi, Hegu
Deng Xiao-dong, 2018	Biguan, Xuehai, Xuanzhong, Yanglingquan, Huantiao, Fengshi, Quxu, Fenglong, Zusanli
Du Xin, 2018	Baihui, Taiyang, Fengchi, Zusanli, Zhibian, Xiyan, Yanglingquan, Huantiao, Jiexi, Zhongfeng, Hegu
Feng Sheng-wang, 2016	Lianquan, Yifeng, Fengchi, Wangu, Fengfu, Yamen, Daying, Jinjin
Fu Qin-hui, 2016	Shenting, Baihui, Hegu, Fenglong, Jianyu, Quchi, Waiguan, Yanglingquan, Zusanli, Tianjin, Naohui, Weizhong, Chengshan, Taichong, Shangjuxu, Taixi, Qihai, Shenshu
Fu Xiao-feng, 2019	Huatuojiaji, Renying, Baihui
Gao Ting, 2018	Quchi, Waiguan, Hegu, Zusanli, Houxi, Weizhong, Yanglingquan, Huantiao, Juegu, Kunlun
Huang Wei, 2018	Sishencong, Xuanli, Baihui, Qubin
Jiang Ming, 2018	Baihui, Renzhong, Dicang, Shousanli, Quchi, Neiguan, Hegu, Waiguan, Jianliao, Shaoze, Zusanli, Huantiao, Yanglingquan, Fengshi, Xuehai, Chengshan, Yinlingquan,
Lin Bi-yu, 2018	Hegu, Quchi, Baihui, Shenting, Sishencong, Neiguan, Waiguan, Zusanli, Taichong, Taixi, Fengchi, Jiquan, Chize
Liu Sang, 2018	Zusanli, Yanglingquan, Weizhong, Huantiao, Quchi, Hegu, Xinshu, Geshu, Shenshu, Dazhui, Baihui, Renzhong, Taichong, Neiting, Cuanzhu, Dicang, Futu1, Jiache, Yangbai, Tiantu, Fenglong, Xuehai, Qihai, Taixi, Guanyuan
Peng Shao-kun, 2015	Renzhong, Jianyu, Waiguan, Quchi, Chize, Taixi, Taichong, Weizhong, Huantiao, Neiguan, Sanyinjiao, Shousanli, Hegu, Tianfu, Shaohai, Zusanli, Yongquan, Jiquan, Xuehai, Fenglong
Shi You-jia, 2018	Yongquan, Zusanli, Fenglong, Yanglingquan, Weizhong, Hegu, Neiguan, Shuigou, Quchi, Chize, Sanyinjiao, Baihui
Shi Yun-hua, 2017	Renzhong, Baihui, Sishencong, Yintang, Neiguan, Zusanli, Sanyinjiao, Xuehai, Jiquan, Weizhong, Chize
Song Yi, 2017	Baihui, Taiyang, Fengchi, Jianyu, Quchi, Hegu, Baxie, Zhibian, Huantiao, Xiyan, Yanglingquan, Zusanli, Quxu, Taichong, Fenglong, Taixi
Tang You-bin, 2014	Tianzong, Yangchi, Wangu
Wang Jing, 2019	Baihui, Yintang, Neiguan, Yanglingquan, Taixi, Danshu, Geshu, Yongquan, Xinshu, Shenshu
Xiao Yu, 2013	Renzhong, Jiquan, Tongli, Neiguan, Juegu, Zusanli, Sanyinjiao, Yongquan, Jianyu, Quchi, Jianqia, Waiguan, Houxi, Jianzhen, Lieque, Jianqian, Guanyuan
Xie Xiao-juan, 2018	Sanyinjiao, Shenmen, Sishencong, Shenting, Taichong, Neiguan, Taixi
Xu Lei, 2017	Huatuojiaji, Jianyu, Quchi, Waiguan, Hegu, Huantiao, Futu1, Zusanli, Xuanzhong, Qiuxu, Kunlun
Xu Wan-song, 2017	Renzhong, Baihui, Hegu, Quchi, Neiguan, Waiguan, Sanyinjiao, Zusanli, Yanglingquan
Zhang Liao, 2018	Renzhong, Neiguan, Sanyinjiao, Weizhong, Jiquan, Chize
Zhang Ning-xia, 2010	Jianyu, Quchi, Hegu, Yanglingquan, Yinlingquan, Zusanli, Sanyinjiao
Zhou Min-ya, 2016	Baihui, Sishen, Shenting, Neiguan, Shenmen, Sanyinjiao
Zhou Shu-xin, 2018	Shangjuxu, Zusanli, Taiyang, Yifeng, Qianzheng, Fengchi, Taichong, Cuanzhu, Yingxiang, Sibai, Jingming, Jiache, Chengjiang, Renzhong

**Table 5:** Acupuncture control group.

Routine treatment	Treatment mode
RT1	Drugs that promote the functional recovery of brain tissue, neurotrophic drugs, lower blood pressure, hypoglycemic, rehabilitation training
RT2	Good limb position, rehabilitation training
RT3	Lower blood pressure, hypoglycemic, rehabilitation training
RT4	Antiplatelet aggregation, defibrillating, stabilization of plaques, improvement of cerebral circulation and neuroprotection, rehabilitation training
RT5	Regulation of blood lipids, lower blood pressure, hypoglycemic, rehabilitation training
RT6	Nutrition of nerves, improvement of circulation, dehydration and lowering of intracranial pressure to control blood pressure and regulate blood glucose in patients with hypertension and diabetes mellitus, rehabilitation training
RT7	Anticoagulant, antiplatelet aggregation, free radical scavenging, neuroprotective agents, brain cell protective dose therapy
RT8	Blood pressure, blood glucose, regulating blood lipids, giving antiplatelet aggregation, nutritional nerve, as well as symptomatic treatment, prevention and treatment of complications and other basic treatment
RT9	Hypotension, lipid reduction, antiplatelet aggregation, etc.
RT10	Control of blood pressure, blood glucose, antiplatelet aggregation, oxygen support, anti-infection, dehydration, reduction of intracranial pressure, regulation of water and electrolyte disorders, improvement of cerebral metabolism, craniomagnetic stimulation therapy, etc.
RT11	Control of blood glucose, high intracranial pressure treatment: 20°~30°, 20% mannitol 125 ml/ivdrip or 25% glycerin fructose 250 ml/ivdrip, rehabilitation training
RT12	Neurotrophic drugs, rehabilitation training, etc.

**Table 6:** TCMs combined with routine treatment vs. routine treatment on hemorheological indices.

Hemorheological indices	Number of studies	Study ID	Cases of experimental group	Cases of control group	MD (95% CI)	*Z* value	*P* value
WHV	2	Liu Weicheng 2017, Zhang Kefei 2018	110	108	-0.89 (-1.04, -0.74)	11.56	<0.00001
WLV	2	Liu Weicheng 2017, Zhang Kefei 2018	110	108	-2.30 (-4.24, -0.36)	2.32	0.02
PV	2	Liu Weicheng 2017, Zhang Kefei 2018	110	108	-0.49 (-0.68, -0.31)	5.19	<0.00001
HCT	3	Li Yaorong 2015, Zhao Xiaoli 2017, Bian Yonghong 2017	168	168	-2.65 (-4.71, -0.58)	2.51	0.01

**Table 7:** ACU combined with routine treatment vs. routine treatment on serum immunoglobulin.

Serum immunoglobulin	Number of studies	Study ID	Cases of experimental group	Cases of control group	MD (95% CI)	*Z* value	*P* value
IgA	1	Zhou Shu-xin 2018	60	60	-0.77 (-1.09, -0.45)	4.71	<0.00001
IgG	1	Zhou Shu-xin 2018	60	60	-1.87 (-2.51, -1.23)	5.72	<0.00001
IgM	1	Zhou Shu-xin 2018	60	60	-0.91 (-1.23, -0.59)	5.54	<0.00001

**Table 8:** OTTCM combined with routine treatment vs. routine treatment on the observation index.

Indices	Number of studies	Study ID	Cases of experimental group	Cases of control group	MD (95% CI)	*Z* value	*P* value
CSI	1	Ding Min 2018	40	39	-1.26 (-1.95, -0.57)	3.56	0.0004
MOCA	1	Yan Hongda 2018	45	45	3.39 (1.04, 5.74)	2.83	0.005
MRS	2	Li Chaoming 2018, Yang Haixia 2016	73	73	-0.61 (-0.81, -0.42)	6.08	<0.00001

**Table 9:** ACU combined with routine treatment vs. routine treatment on the swallowing function score.

Indices	Number of studies	Study ID	Cases of experimental group	Cases of control group	MD (95% CI)	*Z* value	*P* value
SSA	1	Chen Dan 2018	30	30	-3.40 (-4.99, -1.81)	4.19	<0.00001
VFSS	2	Chen Dan 2018, Feng Sheng-wang 2016	60	60	2.44 (1.74, 3.14)	6.80	<0.00001
ADL	5	Cai Jingjing 2012, Jiang Ming 2018, Shi Youjia 2018, Tang Youbin 2014, Wang Jing 2019	251	250	14.04 (7.23, 20.86)	4.04	<0.00001

**Table 10:** ACU combined with routine treatment vs. routine treatment on BFGF and VEGF expression levels.

BFGF and VEGF expression levels	Number of studies	Study ID	Cases of experimental group	Cases of control group	MD (95% CI)	*Z* value	*P* value
BFGF	1	Gao Ting 2018	46	46	3.90 (2.86, 4.94])	7.36	<0.00001
VEGF	1	Gao Ting 2018	46	46	272.24 (261.12, 283.36)	47.99	<0.00001

**Table 11:** Egger's test.

Indices		Egger's test	
		*Z* value	*P* value
TER	Total	1.4211	0.1553
	TCMs-TER	1.3117	0.1896
	ACU-TER	0.3884	0.6977
	OTTCM-TER	0.9818	0.3262

BI	Total	1.2143	0.2246
	TCMs-BI	0.4194	0.6749
	ACU-BI	-0.6821	0.4952
	OTTCM-BI	1.5219	0.1280

NIHSS	Total	-2.3936	0.0167
	TCMs-NIHSS	-0.4708	0.6378
	ACU-NIHSS	-1.0771	0.2815
	OTTCM-NIHSS	/	/

hs-CRP	TCMs-hs-CRP	-2.5896	0.0096

## Data Availability

The data used to support the findings of this study are included within the article.

## Abbreviations

hs-CRP: Hypersensitive C-reactive protein
HDL: High-density lipoprotein
LDL: Low-density lipoprotein
NIHSS: National Institutes of Health Stroke Scale
RCTs: Randomized controlled trials
TCM: Traditional Chinese medicine
TER: Total effective rate
TC: Total cholesterol
TG: Triglyceride
WHV: Whole high viscosity
WLV: Whole low viscosity.

## Supplementary Materials

Supplementary Materials International coding of acupoints and GRADE evidence profile.
