# Supplementary material for: Analysis of Clinical Efficacy of Traditional Chinese Medicine in Recovery Stage of Stroke: A Systematic Review and Meta-Analysis
Source: Cardiovasc Ther. 2020 Sep 19;2020:7172052. doi: 10.1155/2020/7172052 (PMC7528130; doi:10.1155/2020/7172052)
Supplement: Supplementary Materials — International coding of acupoints and GRADE evidence profile. [file 7172052.f1.docx]

International coding of acupoints

| Acupoint | International coding | Acupoint | International coding | Acupoint | International coding |
| --- | --- | --- | --- | --- | --- |
| Jianliao | TE14 | Guanyuan | CV4 | Taiyang | EX-HN5 |
| Quchi | LI11 | Qihai | CV6 | Wangu | SI4 |
| Hegu | LI4 | Jianzhen | SI9 | Yamen | GV15 |
| Huantiao | GB30 | Naoshu | SI10 | Daying | ST5 |
| Yanlingquan | GB34 | Bingfeng | SI12 | Jinjin | EX-HN12 |
| Zusanli | ST36 | Tianzong | SI11 | Zhibian | BL54 |
| Xuanzhong | GB39 | Quyuan | SI13 | Xiyan | EX-LE5 |
| Jiexi | ST41 | Jianwaishu | SI14 | Zhongfeng | LR4 |
| Kunlun | BL60 | Jianzhongshu | SI15 | Yifeng | SJ17 |
| Taichong | LR3 | Jianyu | LI15 | Shenting | GV24 |
| Dicang | ST4 | Biguan | ST31 | Waiguan | TE5 |
| Yingxiang | LI20 | Xuehai | SP10 | Tianjin | TE10 |
| Jiachengjiang | EX-HN9 | Fengshi | GB31 | Naohui | TE13 |
| Lianquan | CV23 | Fenglong | ST40 | Weizhong | BL40 |
| Fengchi | GB20 | Baihui | GV20 | Chengshan | BL57 |
| Fengfu | GV16 | Renying | ST9 | Shangjuxu | ST37 |
| Taixi | KI3 | Houxi | SI3 | Sishencong | EX-HN1 |
| Shenshu | BL23 | Juegu |  | Xuanli | GB6 |
| Huatuojiaji |  | Sanyinjiao | SP6 | Qubin | GB7 |
| Renzhong | GV26 | Jiquan | HT1 | Dazhui | GV14 |
| Shousanli | LI10 | Chize | LU5 | Neiting | ST44 |
| Neiguan | PC6 | Xinshu | BL15 | Cuanzhu | BL2 |
| Shaoze | SI1 | Geshu | BL17 | Yangbai | GB14 |
| Tiantu | CV22 | Shaohai | HT3 | Yintang | GV29 |
| Tianfu | LU3 | Yongquan | KI1 | Baxie | EX-UE9 |
| Yangchi | TE4 | Shuigou | GV26 | Shenmen | HT7 |
| Danshu | BL19 | Jianqia | EX-UE12 | Futu | ST32 |
| Tongli | HT5 | Lieque | LU7 | Qiuxu | GB40 |
| Qianzheng | EX-HN10 | Jianqian | EX-UE12 | Sishen | EX-HN1 |
| Jingming | BL1 | Cuanzhu | BL2 | Sibai | ST2 |
| Chengjiang | CV24 |  |  |  |  |

**GRADE evidence profile**

**Author(s)**:

**Question**: TCM combined With routine treatment compared to routine treatment for Stroke

**Setting**:

**Bibliography**: . TCM for Stroke. Cochrane Database of Systematic Reviews [Year], Issue [Issue].

| **Certainty assessment** | | | | | | | **№ of patients** | | **Effect** | | **Certainty** | **Importance** |
| --- | --- | --- | --- | --- | --- | --- | --- | --- | --- | --- | --- | --- |
| **№ of studies** | **Study design** | **Risk of bias** | **Inconsistency** | **Indirectness** | **Imprecision** | **Other considerations** | **TCM combined With routine treatment** | **routine treatment** | **Relative (95% CI)** | **Absolute (95% CI)** |  |  |
| **TER** | | | | | | | | | | | | |
| 36 | randomised trials | not serious | not serious | not serious | not serious | none | 1758/1922 (91.5%) | 1276/1739 (73.4%) | **OR 4.03** (3.30 to 4.91) | **184 more per 1,000** (from 167 more to 197 more) | ⨁⨁⨁⨁ HIGH | CRITICAL |
| **TCMs-TER** | | | | | | | | | | | | |
| 12 | randomised trials | not serious | not serious | not serious | not serious | none | 693/776 (89.3%) | 459/625 (73.4%) | **OR 3.08** (2.27 to 4.18) | **161 more per 1,000** (from 128 more to 186 more) | ⨁⨁⨁⨁ HIGH | CRITICAL |
| **ACU-TER** | | | | | | | | | | | | |
| 18 | randomised trials | not serious | not serious | not serious | not serious | none | 831/894 (93.0%) | 641/865 (74.1%) | **OR 4.60** (3.41 to 6.21) | **188 more per 1,000** (from 166 more to 206 more) | ⨁⨁⨁⨁ HIGH | CRITICAL |
| **OTTCM-TER** | | | | | | | | | | | | |
| 6 | randomised trials | serious ^a^ | not serious | not serious | not serious | none | 234/252 (92.9%) | 176/249 (70.7%) | **OR 5.67** (3.24 to 9.93) | **225 more per 1,000** (from 180 more to 253 more) | ⨁⨁⨁◯ MODERATE | CRITICAL |
| **NIHSS** | | | | | | | | | | | | |
| 21 | randomised trials | not serious | serious ^b^ | not serious | not serious | none | 1069 | 1062 | - | MD **3.28 lower** (3.97 lower to 2.58 lower) | ⨁⨁⨁◯ MODERATE | CRITICAL |
| **TCMs-NIHSS** | | | | | | | | | | | | |
| 11 | randomised trials | not serious | serious ^b^ | not serious | not serious | none | 610 | 610 | - | MD **2.54 lower** (3.2 lower to 1.88 lower) | ⨁⨁⨁◯ MODERATE | CRITICAL |
| **ACU-NIHSS** | | | | | | | | | | | | |
| 8 | randomised trials | not serious | serious ^b^ | not serious | not serious | none | 371 | 364 | - | MD **4.76 lower** (7.22 lower to 2.3 lower) | ⨁⨁⨁◯ MODERATE | CRITICAL |
| **OTTCM-NIHSS** | | | | | | | | | | | | |
| 2 | randomised trials | serious ^a^ | serious ^b^ | not serious | not serious | publication bias strongly suspected ^c^ | 88 | 88 | - | MD **3.4 lower** (7.45 lower to 0.65 higher) | ⨁◯◯◯ VERY LOW | CRITICAL |
| **Barthel** | | | | | | | | | | | | |
| 26 | randomised trials | not serious | serious ^b^ | not serious | not serious | none | 1168 | 1152 | - | MD **11.56 higher** (9.45 higher to 13.67 higher) | ⨁⨁⨁◯ MODERATE | CRITICAL |
| **TCMs-Barthel** | | | | | | | | | | | | |
| 6 | randomised trials | not serious | not serious | not serious | not serious | none | 306 | 306 | - | MD **11.08 higher** (9.85 higher to 12.3 higher) | ⨁⨁⨁⨁ HIGH | CRITICAL |
| **ACU- Barthel** | | | | | | | | | | | | |
| 12 | randomised trials | not serious | serious ^b^ | not serious | not serious | none | 556 | 548 | - | MD **13.27 higher** (9.73 higher to 16.81 higher) | ⨁⨁⨁◯ MODERATE | CRITICAL |
| **OTTCM -Barthel** | | | | | | | | | | | | |
| 8 | randomised trials | not serious | serious ^b^ | not serious | not serious | none | 306 | 298 | - | MD **9.24 higher** (5.57 higher to 12.92 higher) | ⨁⨁⨁◯ MODERATE | CRITICAL |
| **FMA** | | | | | | | | | | | | |
| 24 | randomised trials | not serious | serious ^b^ | not serious | not serious | none | 1028 | 1001 | - | MD **12.33 higher** (9.84 higher to 14.82 higher) | ⨁⨁⨁◯ MODERATE | CRITICAL |
| **ACU - FMA** | | | | | | | | | | | | |
| 13 | randomised trials | not serious | serious ^b^ | not serious | not serious | none | 618 | 606 | - | MD **13 higher** (9.73 higher to 16.26 higher) | ⨁⨁⨁◯ MODERATE | CRITICAL |
| **OTTCM- FMA** | | | | | | | | | | | | |
| 11 | randomised trials | not serious | serious ^b^ | not serious | not serious | none | 410 | 395 | - | MD **11.56 higher** (7.88 higher to 15.24 higher) | ⨁⨁⨁◯ MODERATE | CRITICAL |
| **TC** | | | | | | | | | | | | |
| 7 | randomised trials | not serious | serious ^b^ | not serious | not serious | none | 436 | 416 | - | MD **0.54 lower** (0.8 lower to 0.28 lower) | ⨁⨁⨁◯ MODERATE | CRITICAL |
| **TG** | | | | | | | | | | | | |
| 7 | randomised trials | not serious | serious ^b^ | not serious | not serious | none | 436 | 416 | - | MD **0.48 lower** (0.64 lower to 0.31 lower) | ⨁⨁⨁◯ MODERATE | CRITICAL |
| **LDL** | | | | | | | | | | | | |
| 5 | randomised trials | not serious | serious ^b^ | not serious | not serious | none | 326 | 308 | - | MD **0.81 lower** (1.19 lower to 0.42 lower) | ⨁⨁⨁◯ MODERATE | CRITICAL |
| **HDL** | | | | | | | | | | | | |
| 5 | randomised trials | not serious | serious ^b^ | not serious | not serious | none | 326 | 308 | - | MD **0.24 higher** (0.09 higher to 0.38 higher) | ⨁⨁⨁◯ MODERATE | CRITICAL |
| **WHV** | | | | | | | | | | | | |
| 2 | randomised trials | serious ^a^ | not serious | not serious | not serious | none | 110 | 108 | - | MD **0.89 lower** (1.04 lower to 0.74 lower) | ⨁⨁⨁◯ MODERATE | CRITICAL |
| **WLV** | | | | | | | | | | | | |
| 2 | randomised trials | serious ^a^ | serious ^b^ | not serious | not serious | none | 110 | 108 | - | MD **2.3 lower** (4.24 lower to 0.36 lower) | ⨁⨁◯◯ LOW | CRITICAL |
| **PV** | | | | | | | | | | | | |
| 2 | randomised trials | serious ^a^ | serious ^b^ | not serious | not serious | none | 110 | 108 | - | MD **0.49 lower** (0.68 lower to 0.31 lower) | ⨁⨁◯◯ LOW | CRITICAL |
| **HCT** | | | | | | | | | | | | |
| 3 | randomised trials | serious ^a^ | serious ^b^ | not serious | not serious | none | 168 | 168 | - | MD **2.65 lower** (4.71 lower to 0.58 lower) | ⨁⨁◯◯ LOW | CRITICAL |
| **FIB** | | | | | | | | | | | | |
| 9 | randomised trials | not serious | not serious | not serious | not serious | none | 496 | 479 | - | MD **0.39 lower** (0.49 lower to 0.28 lower) | ⨁⨁⨁⨁ HIGH | CRITICAL |
| **HCY** | | | | | | | | | | | | |
| 7 | randomised trials | not serious | serious ^b^ | not serious | not serious | none | 359 | 360 | - | MD **4.38 lower** (6.13 lower to 2.63 lower) | ⨁⨁⨁◯ MODERATE | CRITICAL |
| **hs-CRP** | | | | | | | | | | | | |
| 5 | randomised trials | not serious | serious ^b^ | not serious | not serious | publication bias strongly suspected ^c^ | 225 | 226 | - | MD **0.78 lower** (1.32 lower to 0.23 lower) | ⨁⨁◯◯ LOW | CRITICAL |
| **IgA** | | | | | | | | | | | | |
| 1 | randomised trials | not serious | serious ^d^ | not serious | not serious | none | 60 | 60 | - | MD **0.77 lower** (1.09 lower to 0.45 lower) | ⨁⨁⨁◯ MODERATE | IMPORTANT |
| **IgG** | | | | | | | | | | | | |
| 1 | randomised trials | not serious | serious ^d^ | not serious | not serious | none | 60 | 60 | - | MD **1.87 lower** (2.51 lower to 1.23 lower) | ⨁⨁⨁◯ MODERATE | IMPORTANT |
| **IgM** | | | | | | | | | | | | |
| 1 | randomised trials | not serious | serious ^d^ | not serious | not serious | none | 60 | 60 | - | MD **0.91 lower** (1.23 lower to 0.59 lower) | ⨁⨁⨁◯ MODERATE | IMPORTANT |
| **BFGF** | | | | | | | | | | | | |
| 1 | randomised trials | not serious | serious ^d^ | not serious | serious ^e^ | none | 46 | 46 | - | MD **3.9 higher** (2.86 higher to 4.94 higher) | ⨁⨁◯◯ LOW | IMPORTANT |
| **VEGF** | | | | | | | | | | | | |
| 1 | randomised trials | not serious | serious ^d^ | not serious | serious ^e^ | none | 46 | 46 | - | MD **272.24 higher** (261.12 higher to 283.36 higher) | ⨁⨁◯◯ LOW | IMPORTANT |
| **VFSS** | | | | | | | | | | | | |
| 2 | randomised trials | serious ^a^ | serious ^b^ | not serious | not serious | none | 60 | 60 | - | MD **2.44 higher** (1.74 higher to 3.14 higher) | ⨁⨁◯◯ LOW | IMPORTANT |
| **SSA** | | | | | | | | | | | | |
| 1 | randomised trials | serious ^a^ | serious ^d^ | not serious | serious ^e^ | none | 30 | 30 | - | MD **3.4 lower** (4.99 lower to 1.81 lower) | ⨁◯◯◯ VERY LOW | IMPORTANT |
| **CSI** | | | | | | | | | | | | |
| 1 | randomised trials | serious ^a^ | serious ^d^ | not serious | serious ^e^ | none | 40 | 39 | - | MD **1.26 lower** (1.95 lower to 0.57 lower) | ⨁◯◯◯ VERY LOW | IMPORTANT |
| **MOCA** | | | | | | | | | | | | |
| 1 | randomised trials | serious ^a^ | not serious | not serious | serious ^e^ | none | 45 | 45 | - | MD **3.39 higher** (1.04 higher to 5.74 higher) | ⨁⨁◯◯ LOW | IMPORTANT |
| **ADL** | | | | | | | | | | | | |
| 5 | randomised trials | not serious | serious ^b^ | not serious | not serious | none | 251 | 250 | - | MD **14.04 higher** (7.23 higher to 20.86 higher) | ⨁⨁⨁◯ MODERATE | IMPORTANT |
| **MRS** | | | | | | | | | | | | |
| 2 | randomised trials | serious ^a^ | serious ^d^ | not serious | not serious | none | 73 | 73 | - | MD **0.61 lower** (0.81 lower to 0.42 lower) | ⨁⨁◯◯ LOW | IMPORTANT |

**CI:** Confidence interval; **OR:** Odds ratio; **MD:** Mean difference

#### Explanations

a. The included studies did not give specific information in terms of blinding and allocation concealment.

b. The presence of considerable heterogeneity in the included studies (I-square value >75%).

c. Strongly suspect publication bias.

d. Heterogeneity test P value is very small.

e. Total number of participants not enough to provide a precise result.
